# Artificial intelligence for brain diseases: A systematic review

**DOI:** 10.1063/5.0011697

**Published:** 2020-10-13

**Authors:** Alice Segato, Aldo Marzullo, Francesco Calimeri, Elena De Momi

**Affiliations:** 1 Department of Electronics, Information and Bioengineering, Politecnico di Milano, Milan 20133, Italy; 2 Department of Mathematics and Computer Science, University of Calabria, Rende 87036, Italy

## Abstract

Artificial intelligence (AI) is a major branch of computer science that is fruitfully used for analyzing complex medical data and extracting meaningful relationships in datasets, for several clinical aims. Specifically, in the brain care domain, several innovative approaches have achieved remarkable results and open new perspectives in terms of diagnosis, planning, and outcome prediction. In this work, we present an overview of different artificial intelligent techniques used in the brain care domain, along with a review of important clinical applications. A systematic and careful literature search in major databases such as Pubmed, Scopus, and Web of Science was carried out using “artificial intelligence” and “brain” as main keywords. Further references were integrated by cross-referencing from key articles. 155 studies out of 2696 were identified, which actually made use of AI algorithms for different purposes (diagnosis, surgical treatment, intra-operative assistance, and postoperative assessment). Artificial neural networks have risen to prominent positions among the most widely used analytical tools. Classic machine learning approaches such as support vector machine and random forest are still widely used. Task-specific algorithms are designed for solving specific problems. Brain images are one of the most used data types. AI has the possibility to improve clinicians' decision-making ability in neuroscience applications. However, major issues still need to be addressed for a better practical use of AI in the brain. To this aim, it is important to both gather comprehensive data and build explainable AI algorithms.

## I. INTRODUCTION

Over the last three decades, hospitals and healthcare systems produced a vast quantity of unstructured data such as Medical Imaging (MI) data, genomic information, and free text and data streams from monitoring devices.^1^ The analysis of such data significantly changed the approaches used by medical experts and practitioners for identifying, understanding, and treating brain pathologies, as well as identifying risks and reactions to therapies.^2^ In particular, MI and MI processing started a revolution in the field; indeed, they paved the way for studying, treating, managing, and predicting diseases in a quick and noninvasive manner. Furthermore, advances in image and image processing technologies led to more and more cost-effective and low-risk analysis.^3^ Computed Tomography (CT), Positron Emission Tomography (PET), and Magnetic Resonance Imaging (MRI), for instance, have revolutionized the study of the brain by allowing doctors to perform noninvasive evaluations of the brain structure and to infer causes of abnormal function due to different diseases.^4,5^

**FIG. 1.**
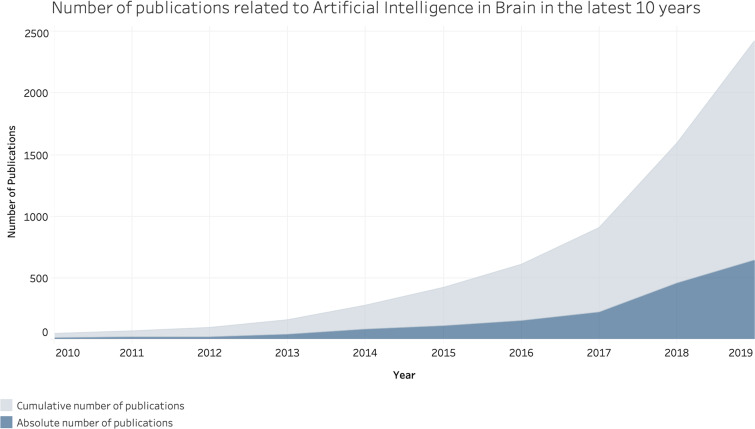
Cumulative and absolute number of papers on artificial intelligence in brain published in the latest ten years (as reported in the considered databases).

However, “manually” processing medical data, and brain images in particular, is often time consuming, and chances of errors in the interpretation are not irrelevant. For example, it has been estimated that day-to-day error rates and discrepancies in radiology are greater than 3%–5%.^6^ This called for novel methods to help physicians in efficiently and effectively analyzing data. As more computational power has been available and the medical data quality increased, the interest in employing advanced algorithms has increased.^7^ However, despite the significant results obtained over the years, given the rise in the complexity and volume of data, many traditional computer-based techniques and algorithms are not feasible in real-world scenarios. For instance, objects like lesions and organs in MI may be too complex to be accurately represented simply by traditional equations or models. Furthermore, it is not always easy for experts to define precise rules to apply, for example, for disease analysis and control. Hence, the use of Artificial Intelligence (AI) techniques has received growing interest in the field of brain imaging and computational neurosciences over the last decade, as demonstrated in the exponential growth of scientific publications reported in Fig. 1. Among these approaches, Machine Learning (ML) techniques are now renowned and widely used for addressing brain-related problems.

ML is a subset of AI algorithms that automatically “learn” to identify categories or forecast future or unknown conditions starting from data. Several solutions have been developed over the years, and many of them still provide successful results in the analysis and processing of brain data.

Quantitative and qualitative characterization studies of normal and pathological structures are often part of clinical tasks in which ML has achieved the most promising results.^8–10^ In this context, brain data processing using ML methods has been widely used to identify brain conditions such as Alzheimer's disease, dementia, schizophrenia, multiple sclerosis, cancer, and infectious and degenerative diseases. Furthermore, approaches for segmentation and detection of brain structures, as well as pathological tissues, are also widely studied.^11^ Detection and precise localization of the abnormal tissue and surrounding healthy structures, indeed, are crucial for diagnosis, surgical planning, postoperative analysis, and chemo/radiotherapy treatment.

Nevertheless, it is worth noting that, because of the complexity and the amount of brain data, ML methodologies usually comprise several steps in order to actually perform a task. For example, image pre-processing, feature selection and ranking, and dimensionality reduction are often required as initial stages to boost algorithm performances up to adequate levels.^12^

**FIG. 2.**
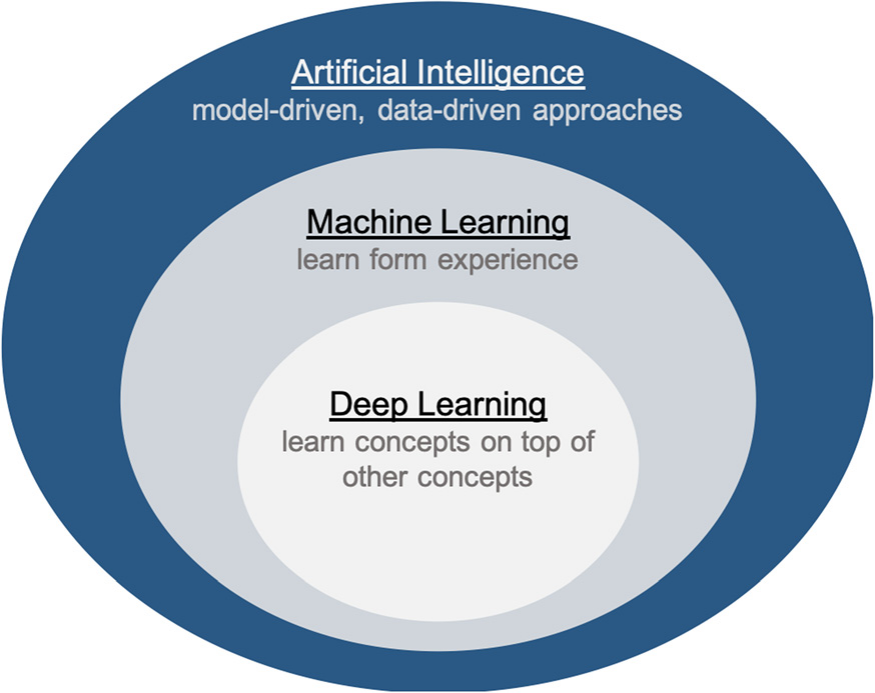
Relations among artificial intelligence, machine learning and deep learning.

In recent years, a subfield of AI, Deep Learning (DL), has revolutionized a variety of neurosurgical tasks^10,13,14^ (Fig. 2). In particular, DL algorithms rose a prominent position in computer vision, outperforming other methods on several high-profile image analysis benchmarks.^15^ Different from traditional ML models, in DL, useful representations and features are learnt automatically, directly from raw data, overcoming the issue of manually computing and selecting potentially relevant attributes. Thanks to critical advancement in computing power, including the use of a Graphic Processing Unit (GPU), such algorithms started to be effectively used for learning from 3D and 2D images typical of the medical domain.^12^

This work primarily focuses on providing an overview of the recent literature on AI techniques directly supporting brain care. We provide a brief analysis of key ideas and areas of application of AI as well as the principal modalities and knowledge used in neuroscience. First, we present a summary of the key clinical uses of AI in the brain, including classification, segmentation, organizational preparation, postoperative analysis, and predictive methods; furthermore, we provide a thorough description of recent classification methods based on brain connectivity; eventually, taking into account recent developments and the rapidly growing potential of the field, we discuss how AI might transform brain care in the near and long term, identifying open issues and promising directions for future work.

The remainder of this paper is organized as follows. We briefly introduce the main type of data used for brain analysis, as well as the main AI techniques adopted for solving brain-related tasks. The methodology used for evaluating the state-of-the-art is explained in Sec. V and analyzed more in detail in Sec. VII. We discuss our findings in Sec. VIII and draw our conclusions in Sec. IX.

## II. TYPE OF DATA

Many different technologies have been developed with the aim of understanding brain structure without the need for invasive neurosurgery. CT and MRI are the two primary innovations that improved diagnostic and management efficiency across the spectrum of neurological disorders.^8,12^ CT uses computer-processed adaptations of several x-ray measurements taken from various angles to produce cross-sectional (tomographic) images. PET is used to observe metabolic processes at cellular levels. MRI uses a strong magnetic field and radio waves to render high-quality imagery of biological structures.^5^

By controlling the radio frequency pulses and the oscillations of the gradient, specific pulse sequences determine how the image is obtained (weighed) and how the different tissues appear. T1- and T2-weighted imagery is useful for demonstrating the anatomy and pathology of the brain, respectively. A third commonly used sequence is the Fluid Attenuated Inversion Recovery (FLAIR).

Advanced imaging is playing an increasingly more important role in the management of patients with neuro-oncologic disease. In this way, advances in Diffusion Tensor Imaging (DTI) and Functional Magnetic Resonance Imaging (fMRI) provide noninvasive means of brain mapping.^16^ More in detail, DTI provides *in vivo* visualization of white matter tracts in the brain, helping to analyze pathological alterations outside visible lesions on MRI.^17^ This is achieved through the creation of a map of the axonal network in the brain by measuring the diffusivity of water molecules. fMRI is a technique to detect eloquent cortex by identifying increased blood oxygen levels in areas of the brain, which are activated by task-based paradigms. During the last two decades, an explosion of fMRI studies took place mapping neural functions to distinct parts of the brain at rest or during task performance. However, much attention has been directed toward Resting State Functional Magnetic Resonance Imaging (rs-fMRI) data.^18^

Hyperspectral Imaging (HSI) is an emerging imaging modality for medical applications, especially in disease diagnosis and image-guided surgery. It provides diagnostic information about the tissue physiology, morphology, and composition. HSI acquires a three-dimensional dataset called hypercube, with two spatial dimensions and one spectral dimension.^19^ Another technique that gained interest because of the capabilities of obtaining real-time visualizations is Intra-operative Ultrasound (IUS), a diagnostic imaging tool that uses high-frequency sound waves to create images of structures in the body.^20^ Ultrasound images are captured in real time using an external probe and ultrasound gel placed directly on the skin.

A fundamental concept in modern neuroscience is that anatomical and functional links between brain regions are arranged in such a way that information processing is close to optimal. Recently applied in neurosciences, graph-based models opened up new perspectives for the study of brain structural and functional integration through graph-derived metrics.^21,22^ In this context, brain connectivity analysis rests upon three different but related forms of connectivity: Structural Connectivity (SC) consists of nodes, corresponding to segmented cortical regions, and links, e constructed by tractography from white matter fiber-tracts.^23,24^ Functional Connectivity (FC), instead, is defined as the temporal dependency of neuronal activation patterns of anatomically separated brain regions. Other brain connectivity forms exist, which, however, are not treated in this review.

Other types involved in the brain care are gene sequence, Electronic Health Record (EHR), Electroencephalography (EEG), and Microelectrode Recording (MER) data. EHR is digitizing valuable medical data on a massive scale. Electronic health records (EHR) capture “real-world” disease and care processes and, hence, offer richer and more generalizable data for comparative effectiveness research than traditional randomized clinical trial studies. EEG measures the weak electromagnetic signals generated by in-brain neuronal activities. It captures both slowly and rapidly changing dynamics of brain activations with a time resolution of milliseconds. This enables the investigation of neuronal activity over a wide range of frequencies that can offer potentially complementary insights regarding how the brain works as a large system.^25^ The MER technique further enhances the ability of the surgeon subcortical area; MER data are used as an adjunct approach to ensure that the Deep Brain Stimulation (DBS) electrode is correctly placed within the target structure.^26^

All input data of the reviewed article are reported in Table I.

**TABLE I.** Type of data explained.

Type of data	Definition
Medical imaging	
MRI (magnetic resonance imaging)	MRI uses a strong magnet and radio frequency (RF) waves to provide clear and detailed pictures of internal organs and tissues.
MRI-T1WI (T1-weighted image)	T1 weighted image is one of the basic pulse sequences in MRI and demonstrates differences in the T1 relaxation times of tissues.
MRI-T2WI (T2-weighted imaging)	T2-weighted image is one of the basic pulse sequences in MRI. The sequence weighting highlights differences in the T2 relaxation time of tissues.
MRI-FLAIR (fluid-attenuated inversion recovery)	FLAIR is an MRI sequence with an inversion recovery set to null fluids.
MRI-DWI (diffusion weighted imaging)	DWI measures the strength of molecular motions of diffusion within a tissue structure or boundaries of white and gray matter brain tissues and brain lesions, which have their own diffusion criteria and can be restricted by the diseases
MRI-DTI (diffusion tensor imaging)	DTI is a magnetic resonance imaging technique that enables the measurement of the restricted diffusion of water in tissue in order to produce neural tract images instead of using these data solely for the purpose of assigning contrast or colors to pixels in a cross-sectional image
PET (positron emission tomography)	PET offers superior soft-tissue contrast and a means of assessing cellular density with diffusion-weighted imaging
CT (computed tomography)	Uses computer-processed adaptations of several X-ray measurements taken from various angles to produce cross-sectional (tomographic) images.
IUS (intra-operative ultrasound)	IUS is a Dynamic imaging modality based on ultrasounds, which provides interactive and timely information during surgical procedures.
fNIRS (functional near-infrared spectroscopy)	fNIRS is a Noninvasive optical imaging technique used to monitor changes in hemoglobin (Hb) amounts within the brain by means of the characteristic absorption spectra of Hb in the near-infrared range.
HSI (hyperspectral imaging)	HSI is an imaging techniques based on capturing and processing of an image using information from all over the electromagnetic spectrum.
Connectivity	
FC (functional connectivity)	FC is a network representing temporal dependency of neuronal activation patterns of anatomically separated brain regions.
SC (structural connectivity)	SC is a network representing anatomical brain regions connected each other through fiber bundles.
Other data	
MER (microelectrode recording)	MER is a technique used for recording electrical patterns from surrounding brain structures.
EEG (electroencephalography)	EEG is a technique for recording and interpreting the electrical activity of the brain.
Gene sequence	Gene sequence is a string of data representing the order of nucleotides in DNA.
EHR (electronic health record)	EHR is digital version of a patient's paper chart.

## III. ARTIFICIAL INTELLIGENCE, MACHINE LEARNING, AND DEEP LEARNING

One of the main AI goals is the development of software for computers or computer-controlled machines able to perform tasks commonly associated with intelligent beings.^1^ Its use in healthcare commonly attempts to emulate and even overcome human cognition in the analysis of complicated medical data.

As schematized in Fig. 2 among the various AI branches, ML plays a prominent role in brain data analysis. ML is an adaptive process that enables computers to learn from experience, learn by example, and learn by analogy.^27^ The goal is to define generic algorithms able to automatically improve their performance over time on the basis of previous results and is achieved by training the algorithms via proper optimization approaches. One of the most valuable properties of such models is the capability of achieving accurate results on several tasks, such as classification or prediction, over unseen data, thus generalizing their learned expertise. In general, every ML algorithm falls into one of the two main categories: supervised learning and unsupervised learning. Supervised learning is generally used when the answer to the problem is known. In this scenario, a set of samples with known labels (training set) is provided to the ML algorithm. Thus, a model is prepared through a training process where its parameters are tuned to produce accurate predictions for the labeled data. Classification methods fall in this category. With unsupervised learning, different from supervised learning, input data are not labeled and no known result is provided to the model. In this case, the algorithm is generally trained at deducing structures and common patterns present in the input data. Clustering is a prime example.

In this review, we mainly focus on the supervised approach, as widely adopted in brain image processing tasks. In this context, several ML solutions provide promising and reliable solutions. According to the function used to process the input, they can be classified into many categories. Among the most common we find Decision Tree (DT), which predicts the output *Y* based on a sequence of splits in the input feature space *X*. Ensembles of DT, such as Random Forest (RF) or boosted trees (e.g., AdaBoost), are thus a more popular choice in most applications since they yield much better prediction performance. Support Vector Machines (SVMs) search for an optimal separating hyperplane between classes, which maximizes the margin, i.e., the distance from the hyperplane to points closest to it on either side.

Among the various ML solutions, Deep Neural Networks (DNNs) are nowadays considered as the state-of-the-art solution for many problems, including tasks on brain images. Such human brain-inspired algorithms have been proven to be capable of extracting highly meaningful statistical patterns from large-scale and high-dimensional datasets. A DNN is a DL algorithm aiming to approximate some function $f^{*}$. For example, a classifier can be seen as a function $y=f^{*}(x,\theta )$ mapping a given input *x* to a category labeled as *y*. *θ* is the vector of parameters that the model learns in order to make the best approximation of $f^{*}$. Artificial Neural Networks (ANNs) are built out of a densely interconnected set of simple units, where each unit takes a number of real-valued inputs (possibly the outputs of other units) and produces a single real-valued output (which may become the input to many other units). DNNs are called networks because they are typically represented by composing together many functions. The overall length of the chain gives the depth of the model; from this terminology, the name “deep learning” arises. Recently, more advanced neural network models with local receptive fields, like Convolutional Neural Networks (CNNs), have proven to have promising classification accuracy in image processing tasks such as classification or segmentation. CNNs replace the fully connected operations by convolutions with a set of “learnable” filters. Success of this approach stems from its ability to exploit the full resolution of 2D and 3D spatial structures (e.g., MRI) without the need for learning too many model parameters, thanks to the weight sharing. Many other DL architectures have been presented over the years; here, it is worth mentioning that the Recurrent Neural Network (RNN) is widely used where longitudinal data are available and the Graph Neural Network (GNN) extends neural networks with the purpose of processing graph structure data.

## IV. CLINICAL AIMS

AI is a major branch of computer science; it counts many methods for building effective tools for analyzing complex domains, including medical data. Its potential to exploit meaningful relationships within a dataset can be used in diagnosis, surgical treatment, intra-operative assistance, and postoperative predicting outcome in many clinical scenarios. Indeed, modern medicine is faced with the challenge of acquiring, analyzing, and applying a large amount of knowledge necessary to solve complex clinical problems. The development of medical AI has naturally been related to the development of AI techniques; in the brain care, these are intended to support healthcare workers in their duties, especially with tasks that rely on the manipulation of data and knowledge. More specifically, in the context of brain care, one of the main purposes is to help clinicians in the formulation of diagnosis “classification” problems, using anatomical, morphological, and connectivity information.^7,8,12,18^ Usually, automatic classification helps clinical decision-making on a pathology of the brain or multiple classes of it, by discerning patterns corresponding to classes. For example, classification methods, using anatomical information, are widely used for the detection of Alzheimer's Disease (AD) and other cognitive impairments,^8^ as well as the characterization of various brain tissues including brain tumors.^14^ Moreover, a classification using morphological information is performed, and the task is known as “image segmentation.”^11^ The goal is to partition an image into multiple regions that share similar attributes, enabling localization and quantification. Segmentation is commonly used for detecting, measuring, and analyzing the main morphological structures of the brain and eventually identifying pathological regions. This accurate structural classification is particularly important in patients with tumors, edema, and necrotic tissues. Brain image segmentation is also useful in clinical diagnosis of neurodegenerative and psychiatric disorders, treatment evaluation, and surgical planning.

To help the formulation of the surgical treatment, similarly, classification is used for surgical candidate selection and segmentation is used for finding and categorizing the surgical target. In brain images, ML detection techniques are performed to identify the areas where the patient's lesions are located as box coordinates and localization of stimulation zones within the brain for DBS treatment used for brain lesion and Parkinson patients. Moreover, AI systems are used for assisting a surgeon during the definition of an optimal trajectory.

Prognosis is extremely important in planning appropriate postoperative treatment. Accurate identification of high-risk patients may facilitate targeted aggressive adjuvant therapy, which may help cure the disease and prolong survival.^28^ The implementation of EHR in hospitals is increasing rapidly; the generated data can be fed to an AI algorithm in their raw form, and the algorithm can try to learn which features are associated with the outcome of interest.^29^ In this way, the algorithm can be able to predict mortality, postoperative hospitalization, transsphenoidal surgery response, DBS outcome, reperfusion, and disease recurrency in a variety of disease conditions including Cushing's disease, Parkinson's disease, brain tumor, brain injury, brain lesion, and neurological disorders, easing the burden of clinicians who have to come up with meaningful structured data.

## V. METHODS

A systematic literature review was performed according to the Preferred Reporting Items for Systematic Reviews and Meta-Analyses (PRISMA) guidelines. In particular, Pubmed, Scopus, and Web of Science databases were searched to identify all potentially relevant studies back to January 1, 2008. The search queries were carefully built with the guidance of a professional librarian using search terms related to artificial intelligence and brain. A comprehensive list of the keywords used for the search is reported in Table II. All biomedical studies that evaluated AI models assisting in brain care were included; duplicates are discarded by using the EndNote reference management software. Following the elimination of duplicates, a careful screening of titles and abstracts was made in order to identify papers that were relevant to our research topic. Any work that matched at least one of the following exclusion criteria was crossed out:

(i) No full-text available
(ii) No AI application
(iii) Conference abstracts
(iv) Animal models
(v) Conference papers
(vi) Books
(vii) Book chapters
(viii) Non-English language.

**TABLE II.** Keywords for the systematic review.

Database	Query
SCOPUS	TITLE-ABS-KEY ( “Machine Learning” AND “Deep Learning” AND ( “Classification” OR “Detection” OR “Identification” OR “Diagnosis” ) AND ( “brain disease” OR “neuro” OR “MRI” OR “medical imaging” ) ) AND ( LIMIT-TO ( DOCTYPE, “ar” ) OR LIMIT-TO ( DOCTYPE, “cp” ) ) AND ( LIMIT-TO ( LANGUAGE, “English” ) ) AND ( LIMIT-TO ( EXACTKEYWORD, “Brain” ) OR EXCLUDE ( EXACTKEYWORD, “Image Segmentation” ) OR EXCLUDE ( EXACTKEYWORD, “Image Reconstruction” ) OR EXCLUDE ( EXACTKEYWORD, “Connectivity” ) OR EXCLUDE ( EXACTKEYWORD, “Functional” ) )
WEB OF SCIENCE	((“Machine Learning” AND “Deep Learning”) AND (“Classification” OR “Detection” OR “Identification” OR “Diagnosis”) AND (“brain disease” OR “brain disorders” OR “MRI” OR “medical imaging” OR “neuro”) NOT “segmentation” NOT “functional” NOT “connectivity”) AND LANGUAGE: (English) AND DOCUMENT TYPES: (Article OR Proceedings Paper)
PUBMED	(“Machine Learning”[Title/Abstract/MeSH] OR “Deep Learning”[Title/Abstract/MeSH]) AND (“classification”[Title/Abstract] OR “diagnosis”[Title/Abstract] OR “identification”[Title/Abstract] OR “detection”[Title/Abstract]) AND (“Brain”[All Fields] AND “MRI”[All Fields]) NOT “Connectivity”[Title/Abstract/MeSH] NOT “Segmentation”[Title/Abstract/MeSH] NOT “Functional”[Title/Abstract/MeSH] NOT Review[ptyp] AND English[lang]

After a proper check of full texts and references, a total of 154 articles/reviews were identified as eligible and, hence, included into this systematic review. Any article appearing to help our research was included and classified; nevertheless, we decided to not cover papers already covered by previous reviews. Data considered from each study were the following:

(1) Application
(2) Name of the first author
(3) Year of publication
(4) Clinical aim
(5) Pathology
(6) Type of data
(7) Data
(8) AI method
(9) Benchmark measure
(10) Results.

On this basis, we computed the distribution of all published articles within the domains of clinical aim, pathology, ML algorithm, and type of data used as input features.

We considered a quantitative synthesis to be inappropriate, due to the heterogeneity in applications. A qualitative synthesis of results is hence provided next by means of a narrative approach. Concerning classification tasks, given the large amount of publications in the literature and the recent results sublimely analyzed, both quantitatively and qualitatively, by previous surveys, we limited the detailed overview to 50 most cited papers of 2019. Finally, we made a strong distinction between image-based and connectivity-based classification tasks. In fact, given the promising results obtained by these techniques, we find the latter to be an evolving challenge that deserves a thorough analysis.

## VI. EVALUATION METRICS

For all AI applications, and ML is no exception, the performance measurement is an essential task. Benchmark measures used for the evaluation of the reviewed studies are explained in Table III. Accuracy, precision, sensitivity, and specificity are metrics widely used to evaluate performance in ML classification tasks. Accuracy and precision reveal a test's basic reliability, while specificity and sensitivity reveal the likelihood of false negatives (FNs) and false positives (FPs). These parameters are largely used, but, as reported by other reviews,^30^ in some cases, these evaluation metrics might not constitute a realistic measure. For these reasons, several works are starting to extend their evaluations by also reporting the Positive Predictive Value (PPV) and Negative Predictive Value (NPV).^31–35^ The Area Under the Curve (AUC) Receiver Operating Characteristic (ROC) curve is one of the most important evaluation metrics to check or visualize the performance of a ML classification problem. It tells how much model is capable of distinguishing between classes: the higher the AUC, the better the model is at predicting. To make a quantitative evaluation of automatic segmentation results, the frequently used procedure is to determine the overlap with the gold standard that in this field is the manual segmentation by and expert radiologist. Generally, Jaccard Coefficient (JC) or Dice Similarity Index (DSI) is used. It ranges from 0 to 1, ranging from no overlap to perfect overlap. For probabilistic segmentation, the validation metric is AUC. Other validation metrics include Mean Square Error (MSE), Peak Signal-to-Noise Ratio (PSNR), Mean Absolute Distance (MAD), and Housdorff Distance (HDD) values. Regarding path planning problems, the most important evaluation metrics reported are the Center of Mass Distance (CMD), Mean Square Distance (MSD), min Square Distance (mSD), and risk score for the trajectory evaluation and time complexity to evaluate the total time of execution for time-constrained applications. For predictive model, the metrics reported are the error rate, Mean Absolute Error (MAE), and Root Mean Square Error (RMSE) that can be interpreted as a measure of the ratio between the true and predicted values.

**TABLE III.** Benchmark measures explained.

Benchmark measures	Definition
Acc (accuracy)	The proportion of correct predictions among the total No. of predictions (TP + TN)/total population.
Sp (specificity)	The proportion of negatively classified cases among the total No. of negative cases; TN/(TN + FP).
Se (sensitivity)	The proportion of positively classified cases among the total No. of positive cases; TP/(TP + FN);
TP (True positive)	An outcome where the model correctly predicts the positive class.
TN (true negative)	An outcome where the model correctly predicts the negative class.
FP (false positive)	Where you receive a positive result for a test, when you should have received a negative results.
FN (false negative)	Where you receive a negative result for a test, when you should have received positive results.
Error (error rate)	The frequency of errors occurred, defined as “the ratio of total number of data units in error to the total number of data units transmitted.”
Risk (risk score)	Designed to represent an underlying probability of an adverse event denoted *Y* = 1, given a vector of P explaining variables *X* containing measurements of the relevant risk factors.
Time (time complexity)	The computational complexity that describes the amount of time it takes to run an algorithm.
JD (Jaccard coefficient)	Also known as the Intersection over Union and the Jaccard similarity coefficient is a statistic used for gauging the similarity and diversity of sample sets. The Jaccard coefficient measures similarity between finite sample sets and is defined as the size of the intersection divided by the size of the union of the sample sets
DSI (dice similarity index)	Statistics for similarity.
PSNR (peak signal-to-noise ratio)	The ratio between the maximum possible power of a signal and the power of corrupting noise that affects the fidelity of its representation.
MSE (mean square error)	Measures the average of the squares of the errors.
RMSE (root mean square error)	Measures the standard deviation of the residual.
FRDD (fault rate dust detection)	Calculated as FRDD = (TP+FN)/(TP+TN+FP+FN)
PCC (Pearson correlation coefficient)	A measure of the linear correlation between two variables X and Y.
HDD (Hausdorff distance)	Measures how far two subsets of a metric space are from each other.
AUC (area under the curve)	A graphical plot illustrating the sensitivity as a function of “1-specificity” in a binary classifier with a varying discrimination threshold. The area under the curve corresponds to the probability that a binary classifier will rank a randomly chosen positive instance higher than a randomly chosen negative one; range 0 to 1.
MAD (Mean absolute distance)	The average absolute distance between two-surface points.
CMD (center of mass distance)	The distance between two centers of mass of surface points.
MSD (mean distance of the surface point)	The average distance between two-surface points.
mSD (min. distance of the surface point)	The minimum distance between two-surface points.
pFDR (positive false discovery rate)	Can be written as pFDR = E[V /R—R > 0], where V is the number of false positives (Type I error) and R is the number of rejected null hypotheses. The term “positive” describes the fact that we have conditioned on at least one positive finding having occurred.
MAE (mean absolute error)	An average of the absolute errors: $|e_{i}|=|y_{i}$-$x_{i}|$, where *y_i_* is the prediction and *x_i_* the true value.
RMSLE (root mean square logarithmic error)	Measures the ratio between actual and predicted. It is then sqrt (mean (squared logarithmic errors)).
Rec (recall)	Quantifies the number of positive class predictions made out of all positive examples in the dataset. It is calculated as the number of TP divided by the total number of TP and FN.
Pr (precision)	Quantifies the number of positive class predictions that actually belong to the positive class. It is calculated as the ratio of correctly predicted positive examples divided by the total number of positive examples that were predicted.
PPV (positive predictive value)	The probability that subjects with a positive screening test truly have the disease.
NPV (negative predictive value)	The probability that subjects with a negative screening test truly do not have the disease.
FAR (false alarm rate)	The number of false alarms per the total number of warnings or alarms in a given study or situation.
ROC (receiver operating characteristic)	Created by plotting the true positive rate against the false positive rate at various threshold settings.
IGV (intergroup variance)	Variations caused by differences within individual groups.

As a final remark, it is worth mentioning the efforts spent by researchers to validate their methods, in order to reduce the possibility of human error and handle variations in brain data. To this aim, a crucial role is played by validation methods. Cross-validation methods (k-fold, leave-one-out, and leave-one-group out) are still the most valuable approach in this sense. Such methods allow us to better validate ML and DL algorithms, avoiding biases that might be present in a single dataset.

## VII. RESULTS

**FIG. 3.**
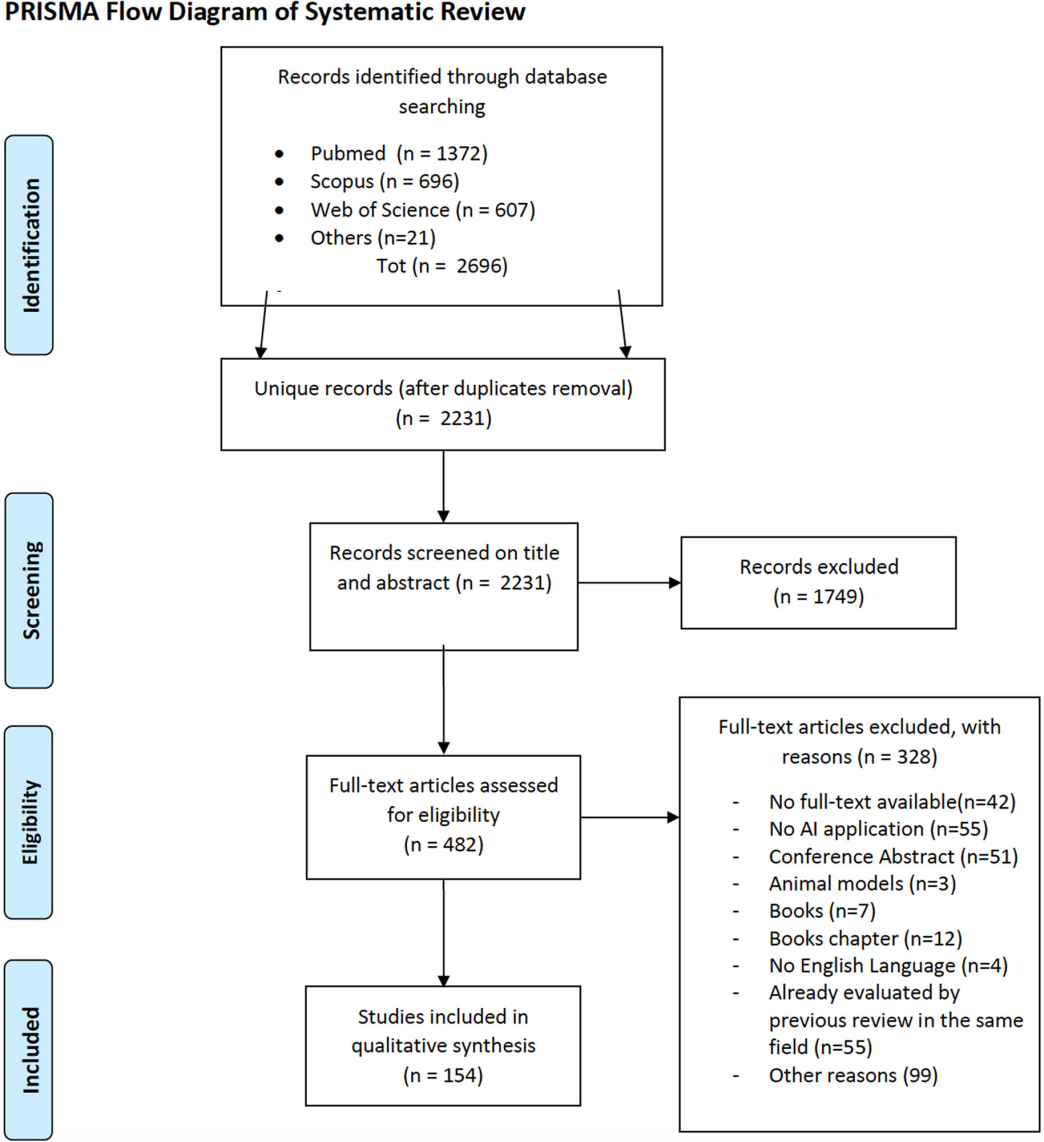
PRISMA flow diagram of systematic identification, screening, eligibility and inclusion. 154 studies were included in the final analysis out of the 2696 screened.

**FIG. 4.**
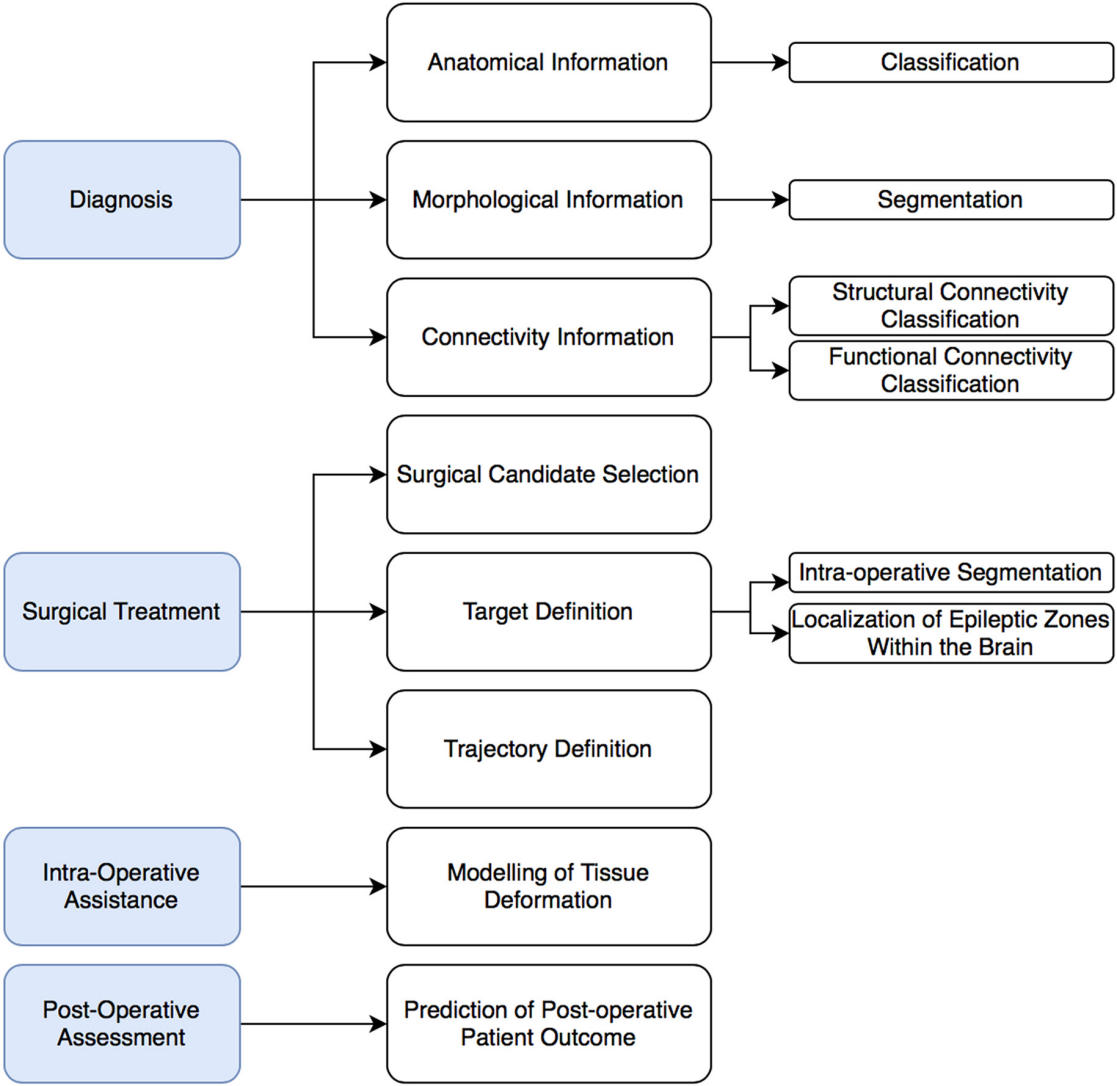
Diagram of multiple paradigms using AI in brain care identified in this review including diagnosis with anatomical information, diagnosis with morphological information, diagnosis with connectivity information, candidate selection for surgical treatment, target definition for surgical treatment, trajectory definition for surgical treatment, modelling of tissue deformation for intra-operative assistance and prediction of patient outcome for postoperative assessment.

Out of the 2696 citations initially identified in the selected databases, 2231 were selected by title/abstract and full-text screening (Fig. 3). We witnessed an exponential growth, in the latest ten years, of the number of studies evaluating AI models as an assisting tool across multiple paradigms of brain care; such paradigms include diagnosis with anatomical information, diagnosis with morphological information, diagnosis with connectivity information, candidate selection for surgical treatment, target definition for surgical treatment, trajectory definition for surgical treatment, modeling of tissue deformation for intra-operative assistance, and prediction of patient outcome for postoperative assessment, as outlined in Fig. 4; AI-enhanced brain care in patients with a wide variety of brain disorders include epilepsy, brain tumors, brain lesion, Parkinson's diseases, brain injury, and cerebrovascular abnormalities. Algorithms used were Natural Language Processing (NLP) algorithms, Genetic Algorithm (GA), ANN, SVM, fuzzy C-means, RF, logistic regression, linear regression, K-nearest Neighbors (KNN), DT, Gradient Boosting Machine (GBM), Sparse Autoencoder (SAE), and k-means, all described in Table IV. A trend in adopting custom solutions, as well as less widely used ML algorithm, was also observed. Commonly used Types of data were MRI, CT, IUS, DTI, HSI, EHR, MER, EEG, and Functional Near-Infrared Spectroscopy (fNIRS). MRI data were the most frequently used input features. Radiological brain tumor segmentation and classification were the most frequently evaluated applications.

**TABLE IV.** AI algorithm explained.

AI algorithm	Mechanism
Regression algorithms	Regression is concerned with modeling the relationship between variables, which is iteratively refined using a measure of error in the predictions made by the model.
Linear regression	Relationships between variables are modeled by fitting a linear equation to observed data.
Logistic regression	Explains the relationship between one dependent binary variable and one or more independent variable regressing for the probability of a categorical outcome using a logistic function.
Instance-based algorithms	An instance-based learning model is a decision problem with instances or examples of training data that are deemed important or required for the model. Such methods typically build up a database of example data and compare new data with the database using a similarity measure in order to find the best match and make a prediction.
KNN (k-nearest neighbor)	Categorize instances based on their similarity with the neighborhood, defined using a proper similarity function (e.g., Eulidean distance).
SVM (support vector machines)	Search for an optimal separating hyperplane between classes, which maximizes the margin, i.e., the distance from the hyperplane to points closest to it on either side.
Bayesian algorithms	Bayesian methods are those that explicitly apply Bayes' Theorem for problems such as classification and regression.
NB (naive Bayes)	Apply Bayes' theorem with the naive assumption of conditional independence between the features.
Clustering algorithms	Clustering, like regression, describes the class of problem and the class of methods.
K-means	By following an iterative procedure, the algorithm creates *K* partitions and assigns entry points to each partition using some heuristic (e.g., similarity with a representative point called the centroid).
Fuzzy C-means	Allows one piece of data to belong to two or more clusters. The procedure is carried out through an iterative optimization of an objective function, with the update of the membership of each data point in each cluster.
HMM (hidden Markov model)	A Markov chain in which states are not directly observable.
SAE (sparse autoencoder)	DNN models trained at reproducing their inputs. Using a proper loss function, the model is forced to rely on a small number of neurons (sparsity).
Artificial neural network algorithms	Artificial neural networks are models that are inspired by the structure and/or function of biological neural networks. They are a class of pattern matching that are commonly used for regression and classification problems but are really an enormous subfield composed of hundreds of algorithms and variations for all manner of problem types.
ANN (artificial neural network)	Network of highly interconnected processing units, which process information by their dynamic state response to external inputs
Deep learning algorithms	Modern update to artificial neural networks that exploit abundant cheap computation. They are concerned with building much larger and more complex neural networks, and, as commented on above, many methods are concerned with very large datasets of labeled analog data
FCNN (fully connected neural network)	ANN in which each unit in a layer is connected with all the units in the next layer.
CNN (convolutional neural network)	ANN in which the fully connected operations by convolutions with a set of learnable filters.
CLNet (corrective learning network)	Explicitly learn a mapping from a new speech segment and the current predictions, to a correction
RNN (recurrent neural networks)	Allows you to model a temporal dynamic behavior dependent on the information received at the previous instants of time by interconnecting higher levels with lower levels.
RFNN (recurrent fuzzy neural networks)	Finds the parameters of a fuzzy system (i.e., fuzzy sets and fuzzy rules) by exploiting approximation techniques from neural networks.
LSTM (long short-term memory networks)	Special kind of RNN, capable of learning long-term dependencies.
DBN (deep belief networks)	Stack of restricted Boltzmann machines, where the nodes in each layer are connected to all the nodes in the previous and subsequent layer.
ELM (extreme learning machines)	Single hidden layer NN where the weights between inputs and hidden nodes are randomly assigned and remain constant during training and predicting phases.
Dimensionality reduction algorithms	Like clustering methods, dimensionality reduction seeks and exploits the inherent structure in the data, but in this case in an unsupervised manner or order to summarize or describe data using less information. This can be useful to visualize dimensional data or to simplify data, which can then be used in a supervised learning method. Many of these methods can be adapted for use in classification and regression.
LDA (linear discriminant analysis)	Projects a dataset of *n*-dimensional samples onto a latent subspace *k* (k≤n−1) while preserving class-discriminatory information.
Ensemble algorithms	Ensemble methods are models composed of multiple weaker models that are independently trained and whose predictions are combined in some way to make the overall prediction.
AdaBoost	The algorithm generates *H* hypotheses through an ensemble of learning algorithms. The output of the learning algorithms is combined into a weighted sum that represents the final output of the boosted classifier.
RF (random forest)	Consists of a large number of individual decision trees that operate as an ensemble. Each individual tree outputs a class prediction and the class with the most votes represents the model's prediction.
GBM (gradient boosting machines)	ML technique providing a prediction model in the form of an ensemble of weak prediction models
GBRT (gradient boosted regression trees)	GBM with decision tree predictors.
Sparse MVTC (sparse multi-view task-centralize)	Multi-view and multi-task ensemble classification method for image-based ASD diagnosis.
Other artificial intelligence algorithms	
GA (genetic algorithm)	A number of candidate solutions (individuals) for a problem are created. The algorithm reflects the process of natural selection where the fittest individuals are selected for reproduction in order to produce offspring of the next generation. Fitness is evaluated by a proper optimization function.
NLP (natural language processing)	Techniques to process and understand the natural language.
GBS (graph-based semisupervision)	Semisupervised learning method in which labeled and unlabeled data are jointly represented as a weighted graph; the resulting graph structure is then used as a constraint during the classification of unlabeled data points.
Multivariate analysis	Involves observation and analysis of more than one statistical outcome variable at a time.
Supervised LOCATE (locally adaptive threshold estimation)	Determines the optimal local thresholds to apply to the estimated lesion probability map, as an alternative option to global thresholding.

### A. Diagnosis

**FIG. 5.**
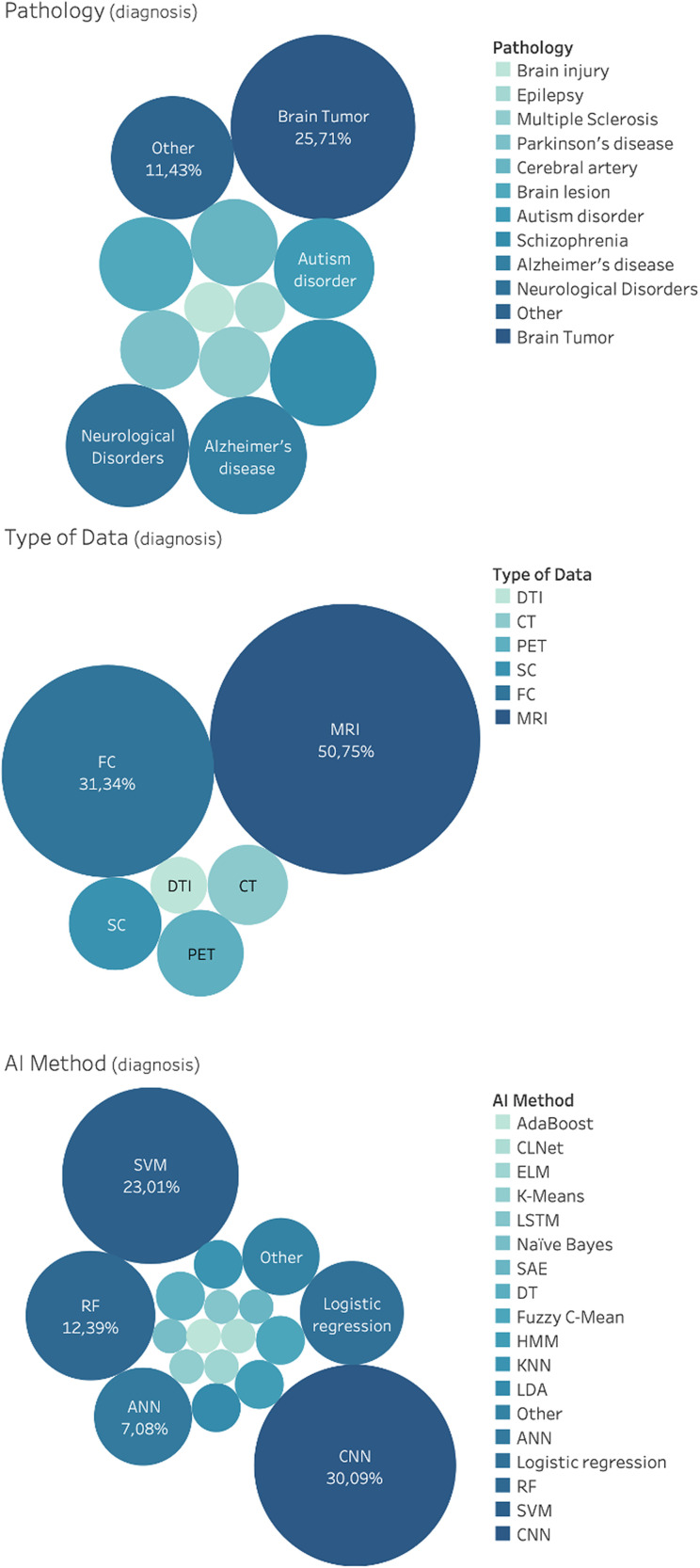
Distribution of general applications for diagnosis, in the brain care literature, related to classification using anatomical information, morphological information and connectivity information. From top: Pathology, AI method and type of data.

121 studies considered AI for diagnosis. This includes classification using anatomical information, morphological information, and connectivity information for neurological disorders, brain tumors, brain lesion, brain injury, Parkinson's disease, epilepsy and cerebral artery, schizophrenia, Alzheimer's disease, autism disorder, and multiple sclerosis. CT, MRI, PET, SC, and FC data were used as input features for the development of classification algorithm. Results of the distributions for pathology, AI methods and type of data for diagnosis are reported in Fig. 5. Notice that, due to the vast heterogeneity of sub-tasks found concerning classification using anatomical information, a qualitative rather than a quantitative research design was chosen.

Computer-Assisted Diagnostics (CAD) reflects a large portion of the various facets of AI for medical imaging.^12^ Such tools constitute a valuable resource for the assistance of medical doctors in diagnosis, prognosis, and pre-and postsurgical processes. One key task is to automatically determine the presence or absence of a disease or a particular type of malignancy.^36–41^ This classification stage is focused on making clinical decisions on a pathology of the brain or multiple classes of it, by discerning patterns corresponding to classes.^42–52^

Several ML-based algorithms have been proposed in recent years for automatically discovering and exploiting visual characteristics statistically associated with clinical outcomes.^5^ Specifically, as previously observed by related studies,^5,7,12,53^ a variety of suitable solutions, mainly based on supervised learning techniques, have been developed for addressing classification tasks in brain imaging.^54–57^ As observable from Table V, different works still exploit classic ML algorithms such as the SVM and its variants^58–62^ and RF.^63–65^ Such algorithms, indeed, provide desirable characteristics, especially for the clinical domain. Other than achieving accurate solutions, indeed, their capability to quantify feature importance measures forms the basis for their explainability.

**TABLE V.** Anatomical information: details on the clinical aim, type of data, dataset, AI method, benchmark measure, and results.

First author (year of publication)	Clinical aim	Pathology/anatomical area	Type of data	Dataset	AI method(s)	Benchmark measure	Results
Anatomical information							
Pang (2019)^69^	Classification of various brain disorders	Neurological disorders	CT MRI	…	CNN SVM RF	Acc	…
Talo (2019)^36^	Classification of various brain disorders	Neurological disorders	MRI-T2WI	…	CNN	Acc	…
Spiteri (2019)^62^	Cerebellar mutism syndrome identification	Neurological disorders	MRI	40	SVM	AUC ROC	…
Squarcina (2019)^190^	Bipolar disorder classification	Neurological disorders	MRI-T1WI	75	Graph-based semisupervision	Acc Sp Se AUC	…
Ramasubbu (2019)^37^	Depression Disorder classification	Neurological disorders	MRI-T1WI clinical	44	SVM	Acc Sp Se	…
Zhou (2019)^70^	AD vs MCI classification	Alzheimer's disease	MRI PET Gene sequence	805	DNN	Acc	…
Basaia (2019)^191^	AD vs MCI classification	Alzheimer's disease	MRI-T1WI	1409	CNN	Acc	…
Spasov (2019)^42^	AD vs MCI classification	Alzheimer's disease	MRI-T1WI	785	CNN	Acc AUC	…
Wang (2019)^68^	AD vs MCI classification	Alzheimer's disease	MRI-T1WI	624	CNN	Acc Pr Rec DSI	…
Mehdipour (2019)^192^	AD progression modeling	Alzheimer's disease	MRI-T1WI	742	LSTM	AUC ROC	…
Bohle (2019)^43^	AD classification	Alzheimer's disease	MRI-T1WI	344	CNN	Acc	…
Martinez-Murcia (2020)^83^	AD diagnosis	Alzheimer's disease	MRI-MRI-T1WIWI	479	CNN SVM	Se Sp Acc DSI	…
Raza (2019)^44^	AD diagnosis	Alzheimer's disease	MRI	432	CNN SVM	Acc Rec Pr Se Sp	…
Wang (2019)^66^	MCI vs AD classification	Alzheimer's disease	MRI-T1WI	624	CNN	Acc Pr Rec DSI	…
Yamashita (2019)^60^	AD diagnosis	Alzheimer's disease	PET MRI	507	SVM	Acc Pr Rec Sp ROC	…
Benyoussef (2019)^45^	AD diagnosis	Alzheimer's disease	MRI	416	CNN KNN	…	…
Forouzannezhad (2019)^41^	MCI diagnosis	Alzheimer's disease	MRI PET Clinical	…	CNN	Acc Sp Se	…
Jabason (2019)^46^	AD diagnosis	Alzheimer's disease	MRI-T1WI	…	CNN	Acc Se Sp	…
Khan (2019)^72^	AD diagnosis	Alzheimer's disease	MRI	…	CNN	Acc Pr Rec DSI	…
Punjabi (2019)^47^	AD classification	Alzheimer's disease	MRI-T1WI PET	723	CNN	Acc	…
Kim (2019)^48^	AD vs dementia classification	Alzheimer's disease	MRI-T1WI	339	LDA	Acc Sp Se AUC	…
Sato (2019)^56^	AD classification	Alzheimer's disease	PET	379	CNN	AUC ROC	…
Eitel (2019)^79^	MS diagnosis	MS	MRI-T2WI	147	CNN	Acc AUC	…
Mato-Abad (2019)^49^	MS classification	MS	DWI	34	Naive Bayes DNN	AUC ROC	…
Ebdrup (2019)^65^	Diagnosis of Schizophrenia	Schizophrenia	MRI EHR	104	SVM RF DT	Acc	…
Talpalaru (2019)^50^	Schizophrenia identification	Schizophrenia	MRI-T1WI	167	Logistic regression RF SVM	ROC AUC	…
Kniep (2019)^63^	Metastatic tumor type classification	Brain tumor	MRI-T1WI MRI-FLAIR	189	RF	Acc AUC	…
Kunimatsu (2019)^58^	Tumor type classification	Brain tumor	MRI-T1WI	76	SVM	Se Sp AUC	…
Wu (2019)^64^	Tumor type classification	Brain tumor	MRI	126	RF	Acc AUC	…
Kebir (2019)^57^	Tumor type classification	Brain tumor	MRI PET	39	SVM	ROC AUC	…
Swati (2019)^71^	Tumor type classification	Brain tumor	MRI	233	CNN	Acc Pr Rec Sp DSI	…
Jeong (2019)^39^	Tumor type classification	Brain tumor	MRI-T2WI MRI-FLAIR	25	RF	Acc AUC	…
Pan (2019)^51^	Tumor mutation prediction	Brain tumor	MRI-T1WI MRI-T2WI	151	RF	Acc AUC	…
Sultan (2019)^38^	Tumor type classification	Brain tumor	MRI-T1WI	233 + 73	CNN	Acc Pr Se Sp	…
Ozyurt (2020)^82^	Brain tumor detection	Brain tumor	MRI-T1WI	500	CNN	Acc AUC ROC	…
Ahammed (2019)^40^	Tumor grade identification	Brain tumor	MRI-T2WI	20	CNN	Acc Pr Rec DSI Se Sp	…
Wang (2019)^80^	Diagnosis of liver tumor	Brain tumor	MRI	334	CNN	PPV Se Pr	
	…					Rec	
Shrot (2019)^61^	Tumor type classification	Brain tumor	MRI DTI	141	SVM	Acc Sp Se	…
Rehman (2019)^73^	Tumor type classification	Brain tumor	MRI	233	CNN SVM	Acc Pr Se Sp	…
Tian (2019)^52^	Glioblastoma vs anaplastic astrocytoma classification	Brain tumor	MRI-T1WI	123	LDA	Se Sp AUC	…
Ortiz-Ramon (2019)^54^	Ischemic stroke lesion identification	Brain lesions	MRI-T1WI MRI-T2WI MRI-FLAIR	100	RF SVM	AUC	…
Lau (2019)^55^	WM hypertensity detection	Brain lesions	MRI-T1WI MRI-T2WI MRI-FLAIR	180	DNN	Se Sp AUC	…
Kim (2019)^138^	Ischemic stroke lesion identification	Brain lesions	EHR	…	DT	Acc Pr Rec DSI	…
Shen (2019)^133^	Parkinson's disease diagnosis	Parkinson's disease	PET	350	Deep Belief network	Acc Se Sp	…
Lee (2019)^78^	Hemorrhage detection	Cerebral artery	CT	196	CNN	Se Sp AUC	…
Ker (2019)^67^	Brain hemorrhage detection	Cerebral artery	CT	399	CNN	DSI	…
Dawud (2019)^75^	Hemorrhage classification	Cerebral artery	CT	…	CNN SVM	Acc	…
Liu (2019)^112^	Cerebral microbleed detection	Cerebral artery	MRI	255	CNN	AUC ROC	…
Gunter (2019)^59^	DESH detection	Other	MRI-T1WI	1576	SVM	AUC ROC	…
Xin (2019)^113^	Gender identification	Other	Diffusion MR	1065	CNN	Acc	…

However, it is evident that a lot of work relies on DL solutions. Independent of the clinical aim, several papers have been proposed exploiting CNNs for classification purposes.^7,53^ Thanks to their capability of extracting latent complex patterns, these algorithms still caught the interest of a wide community and constitute the state-of-the-art for many classification tasks. Indeed, medical imaging is usually achieved by taking slices of the tissue to be analyzed; however, given that the body consists of 3D objects in motion, all images need to be interpreted in order to be actually useful. Such data can be initially processed by a 3DCNN, thus reducing the time needed for human evaluation and fostering a faster patient care.^66,67^ Several novel architectures have been proposed, achieving significant performance with overall accuracy greater than 90% for many classification tasks. In the study by Wang *et al.*,^68^ an ensemble of 3D-densely connected CNNs for AD and Mild Cognitive Impairment (MCI) diagnosis was proposed, outperforming previous methods in all four classification tasks. Such kinds of models allow us to process three-dimensional volumes offering a more global and anatomically meaningful view of the input data with respect to the classical bi-dimensional version. Pang *et al.*^69^ introduced a novel fused CNN that combines shallow layer features and deep layer features. In the analysis, it was observed that the shallow layers provided more detailed local features, which could distinguish different diseases in the same category, while the deep layers could convey more high-level semantic information used to classify the diseases among the various categories. In Zhou *et al.*,^70^ the authors aim to maximally utilize multimodality neuroimaging and genetic data for identifying AD and its prodromal status, MCI from normal aging subjects. The proposed approach consists in stage-wise learning latent representations of each modality first independently and then jointly in order to finally learn diagnostic labels.

A widely used technique for enhancing results on limited datasets is transfer learning (also known as fine-tuning or pre-training), which consists in training the model on large banks of natural images, before actually training it over the small (medical) dataset. This technique allows the model to learn general features like shapes, colors, and patterns, which can be used to process the small dataset more effectively.

Several works applied this technique to enhance their results.^71–76^ They show how fine-tuned models achieved state-of-the art results and how the effect of reduction in training data did not impact the performance of the fine-tuned CNN models.

Another possible way to overcome limited data availability is to artificially create new data. As an example, new instance images can be obtained by applying linear transformations (i.e., rotation, reflection, scaling, etc.) to already available ones. One of the most interesting alternatives, when dealing with image data, consists of learning the latent manifold on which the input images lie and then sample realistic pictures (and their labels) from this manifold. Researchers are investigating this approach in the biomedical domain, achieving promising results. For example, the recently proposed Wasserstein-Generative Adversarial Network (GAN) model was applied by Wegmayr *et al.*^77^ to generate a synthetically aged brain image given a baseline image. The aged image is passed to an MCI or AD discriminator deciding the future disease status, achieving 73% accuracy on MCI-to-AD conversion prediction at a 48 months follow-up using only one coronal slice of a patient's baseline T1image.

Another current DL limitation is related to the understanding of the model when performing the decision-making process. In this direction, Lee *et al.*^78^ proposed a DL system to detect acute intra-cranial hemorrhages and classify Intra-cerebral Hemorrhage (ICH) subtypes. The system was equipped with an *attention map* and a prediction basis retrieved from training data in order to enhance explainability. In the study by Bohle *et al.*, Layerwise Relevance Propagation (LRP) was used to produce heatmaps to visualize the importance of each voxel. They showed how this method was very specific for individuals with high interpatient variability. A similar approach was introduced in the study by Eitel *et al.*,^79^ where LRP helped in explaining MS diagnosis by showing relevant brain areas beyond visible lesions. Wang *et al.*^80^ trained a CNN to distinguish six hepatic tumor entities. Interestingly, the presence of previously manually defined features was assessed by analyzing the CNN activation patterns. Lee^81^ proposed a novel framework to better understand which parts of the brain were pathological and how different brain regions are related to symptomatic observations. To this aim, Regional Abnormality Representations were extracted using complex linear relationships among voxels.

Also, “hybrid” approaches are widely adopted.^82^ In a typical CNN architecture, the feature extraction part includes several convolution layers followed by max-pooling and an activation function. Output of these layers provides latent representation of the original input space, which could constitute useful information. In hybrid approaches, features are extracted from the CNN layers and then used to feed a shallow classifier that performs the classification task. To this aim, a widely adopted pipeline consists in encoding input features by means of Autoencoder architectures. In such models, a network is trained to reconstruct its input. This technique is typically used for dimensionality reduction since, in its simplest version, the input is projected in a smaller latent space before being reconstructed. In this scenario, latent encoding is used as input for the shallow classifier, as it contains “compressed” informative content. Martinez-Murcia *et al.*,^83^ for example, extracted high-level abstract features directly from MRI images and performed an exploratory data analysis of AD based on deep convolutional autoencoders. They observed how the imaging-derived markers could predict clinical variables with correlations above 0.6, achieving a classification accuracy over 80% for the diagnosis of AD.

Segmentation can be treated as a pixel- or voxel-level image classification problem. In recent years, several methods have been adapted to segmentation of complex structures, thus producing accurate and robust segmentation. However, the high intersubject variability along with the modifications caused by pathology makes automatic segmentation a very challenging task. Classical ML methods such as the SVM represent a valuable resource in this sense, especially when meaningful features can be manually extracted. However, it is worth observing that CNNs represented a significant breakthrough in the advancement of brain image segmentation. Their effectiveness is based on multiple convolutional and down-sampling layers that extract image features at different scales. The well-known and currently very popular U-Net^84^ architecture and 3DCNN solutions^85–87^ allowed us to achieve cutting-edge results in many brain structure segmentation competitions.^88^

As reported in Table VI, AI methods were used preoperatively for radiologic segmentation, as previously reported in other reviews.^1,29^ Segmentation of the anatomical structure is important for the diagnosis and treatment of many neurological disorders.^89^ Yepes-Calderon *et al.*^90^ presented a segmentation strategy for the cerebral ventricular volume, based on an algorithm that uses four features extracted from the medical images to create a statistical estimator capable of determining ventricular volume. When compared with manual segmentation, the correlation was 94% and holds promise for even better accuracy by incorporating the unlimited data available. Cherukuri *et al.*^91^ used a learning approach that treats segmentation as supervised classification at the pixel level. The proposed algorithm is computationally less burdensome and exhibits a graceful degradation against a number of training samples.

**TABLE VI.** Morphological information: details on the clinical aim, pathology/anatomical area, type of data, dataset, AI method, benchmark measure, and results.

First author (year of publication)	Clinical aim	Pathology/anatomical area	Type of data	Dataset	AI method(s)	Benchmark measure	Results
Morpholoical information							
Yepes (2018)^90^	Determine the quantity of CSF	Neurological disorders	MRI-T1WI	44	SVM	Acc	94%
Cherukuri (2018)^91^	Determine the quantity of CSF	Neurological disorders	CT	15	CNN	Time	0.003 s
Thillaikkarasi (2019)^92^	Early detection of brain tumor	Brain tumor	MRI	40	CNN SVM	Acc Error Time	98% 15% 15 ms
Sharma (2019)^93^	Simulating tissue deformation and locating cancerous nodes	Brain tumor	MRI-T1WI	6	HMM	Acc PSNR MSE FRDD	88% 21 985 mm 72%
Pushpa (2019)^94^	Detect and classify the tumor type	Brain tumor	MRI	60	SVM	Acc	99%
Rundo (2018)^99^	Necrosis extraction of brain tumor	Brain tumor	MRI	32	Fuzzy C-Means	DSI MAD	95.93% 0.22 pixel
Laukamp (2019)^95^	Volumetric assessment of meningiomas	Brain tumor	MRI-T1WI MRI-T2WI	56	CNN FCNN	DSI	81%
Chen (2019)^96^	Detect mutations in aniopharyngioma patients	Brain tumor	MRI-T1WI	44	RF	AUC Acc Sp Se	89% 86% 85%
Soltanine (2018)^97^	Segmentation of brain tumor	Brain tumor	MRI MRI-DTI	30	RF	DSI Se Error	89% 96% 2%
Sengupta (2018)^98^	Segmentation of brain tumor	Brain tumor	MRI-T1WI MRI-T2WI	9	SVM	Error	8.2%
Perkuhn (2018)^100^	Segmentation of brain tumor	Brain tumor	MRI-T1WI MRI-T2WI MRI-FLAIR	64	CNN FCNN	DSI	86%
Liu (2018)^101^	Segmentation of brain tumor	Brain tumor	MRI	…	CNN SVM	DSI Acc	77.03% 94.85%
Fabelo (2018)^102^	Segmentation of brain tumor	Brain tumor	HSI	5	K-means	Acc Se Se	99% 96% 96%
Binaghi (2019)^103^	Segmentation of meningiomas	Brain tumor	MRI-T1WI MRI-T2WI	15	SVM	JD DSI Error	81% 88.9% 21.74%
Sundaresan (2019)^104^	Lesion segmentation	Brain lesions	MRI-T1WI MRI-T2WI MRI-FLAIR	60	Supervised learning LOCATE	DSI	70%
Praveen (2018)^105^	Segmentation of ischemic stroke lesion	Brain lesion	MRI	28	SAE SVM	DSI Sp Acc Se	94.3% 96.8% 90.4% 92.4%
Remedios (2019)^106^	Segmentation of brain injury	Brain injury	CT	…	3 ANN	DSI PCC	64% 87%
Park (2019)^107^	Segmentation for DBS	Parkinson's disease	MRI-T2WI	102	FCNN	DSI Acc JD	90.2% 90.4% 81.3%
Hadar (2018)^108^	Hippocampal segmentation in temporal lobe epilepsy	Epilepsy	MRI-T1WI	47	CLNet	DSI	85%
Li (2020)^109^	Cerebrovascular segmentation	Cerebral artery	MRI-T1WI	109	HMM	DSI	93%
Lee (2019)^110^	AVM identification and quantification	Cerebral artery	MRI-T2WI	39	Fuzzy C-Means	DSI Se Sp	79.5% 73.5% 85.5%

Tumor segmentation is used for neurosurgical planning to extract the three-dimensional shape from an MRI scan and its relationship with the surrounding anatomy. Thillaikkarasi and Saravanan^92^ presented a novel DL algorithm (kernel based CNN) with the M-SVM to segment the tumor automatically and efficiently. Experimental results of the proposed method can show that the presented technique can executes brain tumor segmentation accurately reaching almost 84% in evaluation with existing algorithms. Sharma and Rattan^93^ proposed a method of segmentation based on a statistical model called the Hidden Markov Model (HMM). The results obtained from parametric analysis show that this algorithm has performed better than the technique of Support Vector Regression (SVR) for brain cancer segmentation, in terms of PSNR, MSE, Fault Rate Dust Detection (FRDD), and accuracy. Pushpa and Louies^94^ presented a SVM algorithm to segment the tumor. The proposed method obtained a better accuracy in classifying the malignant tumor (accuracy of 99%) compared to the other existing systems. Laukamp *et al.*^95^ used a multiparametric DL model on routine MRI data in automated detection and segmentation of meningiomas in comparison to manual segmentation. The DL model yielded accurate automated detection and segmentation of meningioma tissue. Chen *et al.*^96^ adopted Random Forest-based feature selection methods to select the most significant features. They developed a reliable MRI-based radiomics approach to perform pathological and molecular diagnosis. Soltaninejad *et al.*^97^ suggested a novel 3D supervoxel based learning method for segmentation of the tumor. The method provides a close match to expert delineation across all tumor grades, leading to a faster and more reproducible method of brain tumor detection and delineation to aid patient management images. The minimum size for supervoxels regarding its parameters and image characteristics cause limitations in segmenting very small volumes, which can be, however, solved by further postprocessing stages. Sengupta *et al.*^98^ presented a semiautomatic method for segmentation between nonenhancing tumor and vasogenic edema, based on an SVM classifier trained on an alternative ground truth to a radiologist's manual delineation of a tumor. The proposed methodology may prove to be a useful tool for pre- and postoperative evaluation of glioma patients. Rundo *et al.*^99^ implemented a novel fully automatic method for necrosis extraction, using the Fuzzy C-Means algorithm, after the gross tumor volume segmentation. This unsupervised ML technique detects and delineates the necrotic regions in also heterogeneous cancers. Perkuhn *et al.*^100^ evaluate a DL-based, automatic glioblastoma tumor segmentation. The proposed approach for automatic segmentation of this kind of tumor proved to be robust on routine clinical processes. In addition, it showed on all tumor compartments a high automatic detection rate and a high accuracy, comparable to inter-related variability, even if the requirement of all four MR input modalities may limit its applicability. Liu *et al.*^101^ combined CNN features and SVM classifier for the segmentation task, joining the capability of the SVM for classification while avoiding the problem of extracting handcrafted features. Experiments demonstrate that the cascaded CNN method achieves a good tumor segmentation result with a high DSI of 77.03%. However, automatically extracted features may limit the explainability of the model. Fabelo *et al.*^102^ obtained the segmentation map via unsupervised clustering employing a Hierarchical K-Means algorithm. It demonstrated that the use of this method can improve the outcomes of the undergoing patient, assisting neurosurgeons in the resection of the brain tumor. Binaghi *et al.*^103^ suggested a fully automatic procedure based on the allied use of the Graph Cut and SVM. Experimental results, obtained by processing in-house collected data, prove that the method is robust and oriented to the use in clinical practice.

Regarding brain lesion segmentation is used for the diagnosis and follow-up treatment. Sundaresan *et al.*^104^ used LOCally Adaptive Threshold Estimation (LOCATE), a supervised method for determining optimal local thresholds to apply to the estimated lesion probability map, as an alternative option to global thresholding. It allowed us to detect more deep lesions and provided better segmentation of periventricular lesion boundaries. Praveen *et al.*^105^ showed that a deep architecture is using SAE layers. The experimental results showed that the proposed approach significantly outperforms the state-of-the-art methods in terms of precision, DC, and recall.

Segmentation is also used for diagnosis and follow-up treatment of brain injury. Remedios *et al.*^106^ used three neural networks to convergence on a CT brain hematoma segmentation task. Resultant lesion masks with the multi-site model attain an average DSI of 0.64, and the automatically segmented hematoma volumes are correlated with those done manually with a Pearson Correlated Coefficient (PCC) of 0.87, corresponding to an 8% and 5% improvement, respectively, over the single-site model counterparts. Nevertheless, the improvement in performance relies on a significant amount of time at training and inference times, which may limit its practical application.

Segmentation is also used to evaluate deep surgical planning targets for DBS. Park *et al.*^107^ developed DL semantic segmentation-based DBS targeting. A Fully Convolutional Neural Network (FCNN) was used to ensure margin identification by semantic segmentation, proving that the accuracy of DL-based semantic segmentation may surpass that of previous methods.

Segmentation is used to evaluate deep surgical planning targets for epilepsy treatment. Hadar *et al.*^108^ implemented automated segmentation through the Corrective Learning Network (CLNet) method. It demonstrates the clinical utility of automated segmentation in the Temporal Lobe Epilepsy (TLE) MR imaging pipeline prior to surgical resection and suggests that further investigation into CLNet-assisted MRI reading could improve clinical outcomes.

Segmentation is used to assess cerebrovascular reconstruction. Li *et al.*^109^ implemented a novel intensity and shape-based Markov statistical modeling for complete cerebrovascular segmentation. To regularize the individual data processes, the Markov regularization parameter is automatically estimated by using a ML algorithm. This method obtained satisfying results in visual and quantitative evaluation. The proposed method is capable of accurate cerebrovascular segmentation. Lee *et al.*^110^ suggested a fully automated segmentation via unsupervised classification with fuzzy c-means clustering to analyze the Arteriovenous Malformation (AVM) nidus on T2-weighted. The automated segmentation algorithm was able to achieve classification of the AVM nidus components with relative accuracy.

Human connectome research has attained growing interest in neuroscience.^18,111^ Computational methods, particularly graph theory-based methods, have recently played an important role in understanding the architecture of brain connectivity because of their notable ability to describe complex brain systems.^22^ Although the graph theoretical approach can generally be applied to either functional or structural connectivity patterns, to date, most articles have concentrated on resting-state functional connectivity. In this context, there has been an increasing trend to identify biological markers for the characterization of various brain disorders, including either cognitive impairments or pathological alterations.^30^ Connectivity features alone offer promising diagnostic biomarkers, even if several studies apply feature selection and ranking techniques in order to reduce their complexity. Graph-theory derived metrics and high-level network organization have also been considered as valuable biomarkers and widely used in several studies.^111^

Concerning the classification of brain disorders, during the last decade, several conventional studies focus on binary classification tasks.^112–126^ They primarily seek to discriminate between patients and Healthy Control (HC), as well as separating patients into different sub-groups according to the different stages of brain disorder progression. However, recent studies have also drawn their attention to multi-class classification problems.

Table VII presents a summary of recent studies concerning brain network-based classification tasks. Among various brain disease and disorders, Alzheimer's disease, autism, and schizophrenia have been the most studied in recent years.^127–137^ However, several studies are also focused on Parkinson's disease, Multiple Sclerosis, and Tourette Syndrome, among others.^138–148^

**TABLE VII.** Connectivity information: details on the clinical aim, pathology/anatomical area, type of data, dataset, AI method, benchmark measure, and results.

First author (year of publication)							
Connectivity information	Clinical aim	Pathology/anatomical area	Type of data	Dataset	AI method(s)	Benchmark measure	Results
Nielsen (2020)^151^	Tourette syndome analysis	Neurological disease	FC	202	SVM	Acc	71%
Hirshfeld-Becker (2019)^114^	Depression diagnosis	Neurological disease	FC (longitudinal)	68	SVM	Acc Se Sp	92% 90% 93%
Liu (2019)^115^	Depression diagnosis	Neurological disease	FC	85	LR	Acc Se Sp AUC	77% 84% 72% 87%
Shao (2019)^116^	Bipolar disorder classification	Neurological disease	FC (longitudinal)	200	SVM	Acc Se Sp	78.13% 82% 75%
DSouza (2019)^117^	HiV-associated disorder analysis	Neurological disease	FC	29	AdaBoost	Acc AUC	79% 84%
Ju (2019)^154^	AD diagnosis	Alzheimer's disease	FC	170	DNN	Acc Se Sp AUC	86.47% 92% 81% 91%
Li (2019)^76^	AD diagnosis	Alzheimer's disease	FC	26 292	SVM	Acc Se Sp AUC	84.6% 92% 79% 0.80
Li (2019)^118^	MCI diagnosis	Alzheimer's disease	FC	73	DNN	Acc Se Sp	80.82% 81% 81%
Song (2019)^132^	AD diagnosis	Alzheimer's disease	FC	30	KNN	Acc Se Sp AUC	96% 94% 1% 98%
Wada (2019)^155^	AD vs dementia classification	Alzheimer's disease	FC	48	CNN	Acc Pr Rec	73% 78% 73%
Qureshi (2019)^157^	AD progression analysis	Alzheimer's disease	FC	133	CNN	Acc Sp Se	92% 95% 70%
Nguyen (2019)^34^	Dementia diagnosis	Alzheimer's disease	FC	95	ELM	Acc Se Sp PPV NPV	89.92% 87% 84% 94% 87.40%
Peraza (2019)^131^	AD diagnosis	Alzheimer's disease	SC	78	SVM	Acc Se Sp AUC	89.07% 79% 99% 78%
Kam (2019)^35^	MCI diagnosis	Alzheimer's disease	FC	49	CNN	Acc Se Sp PPV NPV	73.85% 74% 74% 74.38% 73.79%
Wang (2019)^119^	AD diagnosis	Alzheimer's disease	SC (multimodal)	211	LR	Acc^1^ Acc^1^ Acc^1^	97% 83% 97%
Azarmi (2019)^120^	MS diagnosis	MS	FC	20	SVM	Acc Se Sp	95% 0.88% 100%
Sacca (2019)^32^	MS diagnosis	MS	FC	37	SVM RF	Acc Se Sp PPV NPV	85.7% 100% 67% 60% 100%
Lisowska (2019)^121^	Dementia diagnosis	Dementia	SC	84	SVM	Acc Se Sp AUC	76.88% 67% 78% 76%
Wang (2019)^122^	Autism diagnosis	Autism spectrum disorder	FC	1112	Sparse MVTC	Acc Se Sp AUC	73% 79% 64% 72%
Kazeminejad (2019)^149^	Autism classification	Autism spectrum disorder	FC	816	SVM	Acc Se Sp	95% 97% 95%
Payabvash (2019)^31^	Autism diagnosis	Autism spectrum disorder	SC	47	RF	Acc Sp PPV	75.3% 97% 81.5%
Yamagata (2019)^123^	Autism diagnosis	Autism spectrum disorder	FC	60	LR	Acc AUC	75% 78%
Khosla (2019)^158^	Autism diagnosis	Autism spectrum disorder	FC	387 389 213 163	CNN	Acc AUC	100% 77%
Song (2019)^124^	Autism diagnosis	Autism spectrum disorder	FC	39	LDA	Acc Pr Rec	82.08% 81% 81%
Dekhil (2019)^125^	Autism diagnosis	Autism spectrum disorder	FC	185	RF	Acc Se Sp AUC	81% 85% 79% 0.82
Wang (2019)^126^	Autism diagnosis	Autism spectrum disorder	FC	531	SVM	Acc Se Sp	90.60% 91% 91%
Kalmady (2019)^153^	Schizophrenia diagnosis	Schizophrenia	FC	174	Ensemble Learner	Acc Se Sp Pr	87% 80% 93% 92%
Lei (2019)^150^	Schizophrenia diagnosis	Schizophrenia	FC	747	SVM	Acc	81.74%
Li (2019)^127^	Schizophrenia diagnosis	Schizophrenia	FC	148	LDA	Acc	76.34%
Qureshi (2019)^156^	Schizophrenia diagnosis	Schizophrenia	FC	144	CNN	Acc AUC	98.09% 99%
Phang (2019)^128^	Schizophrenia diagnosis	Schizophrenia	FC (multimodal)	84	CNN	Acc Se Sp Pr	90.37% 91% 90% 92%
Deng (2019)^129^	Schizophrenia diagnosis	Schizophrenia	SC	125	RF	Acc Se Sp AUC	71% 67% 75% 79%
Zhao (2019)^130^	Schizophrenia diagnosis	Schizophrenia	FC SC	283	SVM	Acc Se Sp	91.75% 91% 93%
Rangaprakash (2019)^139^	Neurotrauma analysis	Brain injury	FC	87	SVM	Acc	81.4%
Rubbert (2019)^134^	Parkinson's disease diagnosis	Parkinson's disease	FC	89	LR	Acc Sp Se	76.2% 72% 81%
Baggio (2019)^135^	Parkinson's disease diagnosis	Parkinson's disease	FC	151	SVM	Acc Se Sp	77.17% 80% 77%
Pena-Nogales (2019)^136^	Parkinson's disease progression analysis	Parkinson's disease	SC	51	LR	Acc Se Sp AUC	84% 91% 77% 89%
Bharath (2019)^137^	Epilepsy diagnosis	Epilepsy	FC	132	SVM	Acc Se Sp	97.5% 100% 94%
Nielsen (2019)^140^	Brain maturity prediction	Other	FC	122	SVM Multivariate Analysis	IGV	57%
Zhigalov (2019)^141^	Attentional state classification	Other	FC	24	SVM	Acc^1^ Acc^2^ Acc^3^	62% 62% 55%
Brauchli (2019)^142^	Absolute pitch identification	Other	FC	100	SVM	Acc	71.75%
Fede (2019)^143^	Alcohol use severity classification	Other	SC FC	59 24	Linear Regression	IGV	-
Weis (2019)^152^	Gender classification	Other	FC	434 410 941	SVM	Acc	70.33%
Bidelman (2019)^144^	Age-related hearing loss prediction	Other	FC	32	SVM	Acc AUC DSC	85.7% 88% 86%
Wetherill (2019)^145^	Nicotine use disorder identification	Other	FC	216	SVM	Acc AUC	88.1% 93%
Chen (2019)^33^	Fatigue identification	Other	FC	16	SVM	Acc Pr Se FAR	94.4% 94% 95% 5.7%
Al-Zubaidi (2019)^146^	Metabolic state classification	Other	FC	24	SVM	Acc Se Sp	81% 89% 83%
Shen (2019)^133^	Chronic low back pain analysis	Other	FC	160 37	SVM	Acc^1^ Se^1^ Sp^1^ Acc^2^ Se^2^ Sp^2^	79.3% 83% 74% 67% 72%
Chriskos (2020)^147^	Sleep state classification	Other	FC	23	CNN	Acc Rec	99.85% 100%
Feng (2019)^148^	Prediction of dispositional worry	Other	SC	59	LR	RMSE p	13.65% <0.005

Among ML approaches based on classical ML algorithms, a wide range of classifiers has been applied in the classification of brain disorders. The SVM is so far the most popular method, as also observed in earlier reviews.^18,30^

Many studies are related to schizophrenia, bipolar disorder, autism spectrum disorder, attention, AD, and MCI. Kazeminejad and Sotero,^149^ for example, used graph theoretical metrics of fMRI-based functional connectivity of patients with autism and HC, to inform a SVM. They achieved state-of-the-art results (accuracy 96%), also observing that measures of centrality provide the highest contribution. Lei *et al.*^150^ analyzed topological properties of patients with schizophrenia, comparing them with HC. Connectome-wide connectivity allowed single subject classification of patients and HC (average accuracy 81%) better than both whole-brain images and graph-based metrics. However, the SVM is also widely used in other brain connectivity analysis applications. Saccà *et al.*^32^ used functional connectivity to train the SVM, along with various ML algorithms, to distinguish MS patients and HC. Feature selection was performed to identify the most important variables. The SVM and RF achieved the best results (85.7%). In Ref. 151, the SVM was used to classify patients with Tourette syndrome. They observed successful performance in children and adults separately, which, however, did not generalize across age groups, suggesting that connectivity characteristics are age specific. Weis *et al.*^152^ employed the SVM to assess how accurately participant's sex can be classified based on spatially specific resting state brain connectivity.

Ensemble methods, such as RF or boosted trees, are also a more popular choice in most applications since they yield much better prediction performance. In Ref. 31, the authors used tract-based connectivity metrics from structural connectome to classify children with Autistic Spectrum Disorder (ASD). A high level of accuracy was achieved (75%), also observing reduced density of connection edges in the posterior white matter tracts of children with ASD. In Ref. 153, functional connectivity, along with regional activities over a wide range of different parcellation schemes, was used as input for an ensemble ML algorithm. They outperformed earlier ML models built for diagnosing schizophrenia using rs-fMRI.

DL methods have attracted increasing interest in various areas and have also been applied in the classification of brain disorders. Ju *et al.*^154^ used DL with a functional brain network and clinical relevant text information to make early diagnosis of AD. Specifically, a targeted autoencoder is built to distinguish HC from MCI. The study revealed discriminative brain network features and provided a reliable classifier for AD detection (Accuracy 86%, AUC 0.91). In Ref. 155, a six layer CNN was trained using structural connectivity to classify among patients with AD, Dementia, and HC. A 3D-CNN architecture was used in Ref. 156 for the automated discrimination of schizophrenia based on 3D-ICA based functional connectivity networks, achieving promising results (Accuracy 98%, AUC 0.99). A similar approach was proposed in Ref. 157 for AD detection. A 3D-CNN approach was also used in the study by Khosla *et al.*^158^ for autism classification. An ensemble learning strategy to combine the predictions from models trained on connectivity data extracted using different parcellation schemas was proposed. They observed how ensemble learning with stochastic parcellations outperforms atlas-based models (Accuracy: 72%, AUC: 0.77).

As a final remark, it is worth mentioning the role that graph-based DL models have been playing in recent years. These specific deep convolutional neural network architectures, designed for network structures (such as connectomes), feature meaningful interpretations in terms of network topology and have been successfully experimented in different domains, such as prediction of neurodevelopmental outcomes in preterm infants,^159^ AD progression,^160^ and Multiple Sclerosis classification,^161^ among others. These methods paved the way for more interpretable operations of structured data.

Thus, given these latest results, both classical ML algorithms and DL techniques are effective for diagnosis; notably, high performances can be reached even in the presence of limited datasets. In addition, hybrid approaches also provided remarkable results and can represent an interesting solution, as they can combine the advantages of each group of methods and try to compensate weaknesses of methods with strength points from the other methods.

### B. Surgical treatment

**FIG. 6.**
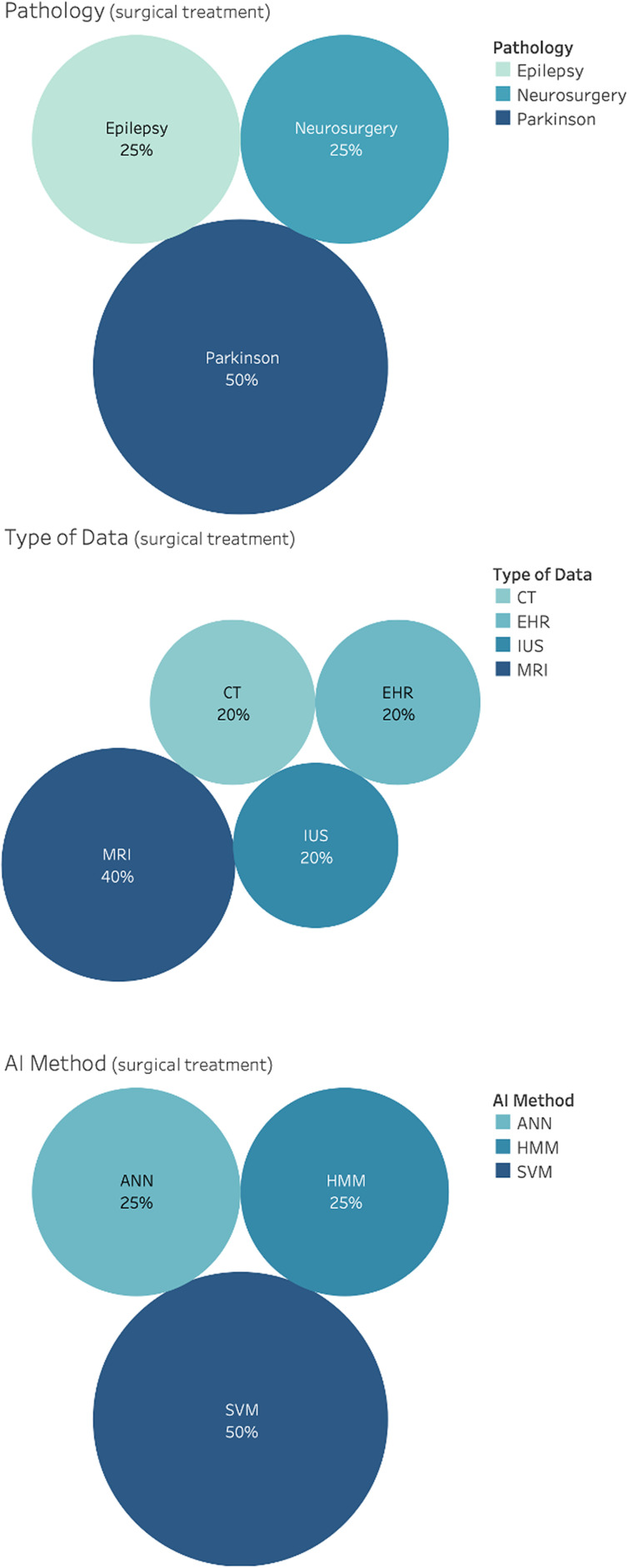
Distribution of general applications for surgical treatment, in the brain care literature, related to target definition and trajectory definition. From top: pathology, AI method and type of data.

18 studies considered AI in surgical treatment. This includes surgical candidate selection, trajectory planning, and target definition with intra-operative segmentation of anatomical structures and localization of stimulation zones for Parkinson's disease, epilepsy, and general neurosurgery. CT, MRI, EHR, IUS, and HSI data were used as input features for the development of the prediction algorithm. Results of the distributions for pathology, AI methods, and types of data for surgical treatment are reported in Fig. 6.

As reported in Table VIII, AI was also useful to identify patients who are potentially eligible for surgery as reported in the review of Senders *et al.*^1^ Wissel *et al.*^162^ validated a NLP application that uses provider notes to assign epilepsy surgery candidacy scores. Currently, NLP represents the state-of-the-art approach for surgical candidate selection, with a specificity of 80% and a sensitivity of 77%. NLP is an AI method for analyzing the unstructured text; an electronic health record-integrated NLP application can accurately assign surgical candidacy scores to patients in a clinical setting. The application learned to assign weights to key words and phrases without needing to incorporate *a priori* domain knowledge. Anyway, although this method may help find patients faster or more comprehensively, its direct effect on surgical outcomes is unknown. Future work should include evaluating the effect of alerting physicians of patients' surgical candidacy scores. As reported in Table IX, AI methods were used for target definition using intra-operative segmentation and localization of stimulation zones within the brain.

**TABLE VIII.** Surgical candidate selection: details on the clinical aim, pathology/anatomical area, type of data, dataset, AI method, benchmark measure, and results.

First author (year of publication)	Clinical aim	Pathology/anatomical area	Type of data	Dataset	AI method(s)	Benchmark measure	Results
Surgical candidate selection							
Wissel (2019)^162^	Candidate selection	Epilepsy	EHR	4211	NLP SVM	AUC Se Sp	80% 77%

**TABLE IX.** Target definition: details on the clinical aim, pathology/anatomical area, type of data, dataset, AI method, benchmark measure, and results.

First author (year of publication)	Clinical aim	Pathology/anatomical area	Type of data	Dataset	AI method(s)	Benchmark measure	Results intra-operative segmentation
Intra-operative Segmentation							
Nitsch (2019)^163^	Segmentation of central ultrasound images	Neurosurgery	IUS MRI	18	ANN	DSI HDD	88% 5.21 mm
Valsky (2019)^164^	Segmentation of striato borders	Parkinson's disease	MER	42	HMM	…	…
Valisky (2017)^165^	Discrimination between the STN and SN	Parkinson's disease	CT MRI	46	SVM	pFDR Time	<0.05% –98%
Localization of epileptic zones within the brain							
Ieong (2019)^167^	localization of lesion due to opiate	Brain lesion	EEG fNIRS	19	SVM GBM ANN	PCC	55%
Wang (2019)^168^	Functional localization	Parkinson's disease	MER	1	K-means SVM KNN	Acc	96%
Shamir (2019)^169^	Visualize STN	Parkinson's disease	MRI	16	RF	Acc	93%
Kim (2019)^170^	Visualize STN	Parkinson's disease	MRI	80	RF	CMD MSD DSI Acc	1.25 mm 0.57 mm 64% 89%
Khosravi (2019)^171^	Visualize STN	Parkinson's disease	MER	50	K-means	Acc	80%
Bermudez (2019)^172^	Localization of the optimal stimulation zone	Parkinson's disease	MRI	187	CNN	AUC	67%
Cimbalnik (2019)^173^	Localization of epileptic foci	Epilepsy	EEG	9	SVM	AUC	<95%
Bharath (2019)^137^	Localization epilepsy network	Epilepsy	MRI-T1WI	42	SVM	Acc Sp Se	97.5% 94.4% 100%

#### 1. Intra-operative segmentation

In intra-operative segmentation for general neurosurgery, Nitsch *et al.*^163^ presented a robust and fully automatic neural-network-based segmentation of central structures of the brain on B-mode IUS. In Intra-operative segmentation for DBS, Valsky *et al.*^164,165^ showed the feasibility of real-time ML classification of striato-pallidal borders and Subthalamic Nucleus (STN) to assist neurosurgeons during DBS surgery. ML algorithms enable real-time Globus Pallidus (GP) and STN navigation systems to potentially shorten the duration of electrophysiological mapping of borders, while ensuring correct border detection. Thus, the ANN represents the state-of-the-art approach for intra-operative segmentation, reaching accuracies of 88%. Compared to a previous method for which a Random Forest classifier was trained with handcrafted features, the Dice coefficient could be increased by 0.14 and the Hausdorff distance is reduced by 7 mm.

#### 2. Localization of stimulation zones within the brain

AI methods were also used for brain lesion and Parkinson patients to localize the stimulation zone and estimate the volume of activated tissue as previously reported in other reviews.^1,166^ Regarding brain lesion stimulation zones, Ieong *et al.*^167^ presented a supervised ML method to obtain associations between EEG and fNIRS modalities to improve precision and localization in assessing neurovascular signals in the prefrontal cortex in opiate addiction patients. Regarding Parkinson's disease stimulation zone, Wang *et al.*^168^ described a functional localization method in the brain. A cubic SVM was used to train the spike pattern recognition model for functional localization with an accuracy of 10% in normal monkey, and the evaluation of the trained model demonstrated a reasonably excellent recognition accuracy of 99.5%. Weighted KNNs showed a better performance of accuracy (94.5%) of spike pattern recognition for functional localization than the cubic SVM. These two works^169,170^ demonstrated that the 7T-ML method is highly consistent with microelectrode-recording data. This method provides a reliable and accurate patient-specific prediction for targeting the STN. Khosravi *et al.*^171^ suggested an unsupervised ML technique to localize the STN during DBS surgery. Bermudez *et al.*^172^ used a patch-based convolutional neural network to classify a stimulation coordinate as having a positive reduction in symptoms during surgery.

Regarding epilepsy stimulation zones, Cimbalnik *et al.*^173^ applied a SVM model for accurate localization of the epileptogenic tissue. The tissue under the iEEG electrodes, classified as epileptogenic, was removed in 17/18 excellent outcome patients and was not entirely resected in 8/10 poor outcome patients. The overall best result was achieved in a subset of 9 excellent outcome patients with the area under the ROC = 0.95. Bharath *et al.*^137^ proposed a SVM to identify and validate the possible existence of the resting state. This approach could classify individuals with epilepsy with 97.5% accuracy, 100% sensitivity, and 94.4% specificity. Thus, given the last results, the SVM represents the state-of-the-art approach for localization of stimulation zones within the brain. In this context, SVM models combine multiple features from rsfMRI epilepsy networks to localize epileptic zones. Interestingly, looking at these results, one can think that other brain networks could also carry disease-sensitive information about epilepsy, as previously illustrated for diagnosis.

As reported in Table X, AI methods were used for the preoperative trajectory definition as reported in the review of Senders *et al.*^29^ AI in general neurosurgery can be used for assisting the surgeon preoperatively for the definition of an optimal trajectory. Briefly, usually, the algorithm aims are to minimize the intra-cerebral catheter length and drilling angle from orthogonal to skull, while maximizing the distance from critical structures. Villanueva-Naquid *et al.*^174^ proposed that the use of a GA drastically reduces the computational cost. Liu *et al.*^175^ used a vector-model-supported optimization for brain tumor surgery. With this approach, there was a significant reduction in the median planning time, a 40% reduction from 3.7 to 2.2 h. Segato *et al.*^176^ presented a GA that drastically reduces the number of trajectories to analyze, speeding up the preoperative planning procedure for DBS in Parkinson patients. In three works,^165,177,178^ two ML approaches, RF and linear regression, were investigated to predict composite ablation scores and determine entry and target point combinations that maximize ablation for Laser Interstitial Thermal Therapy (LITT). RF and linear regression predictions had a high correlation with the calculated values in the test set for both methods.

**TABLE X.** Trajectory definition: details on the clinical aim, pathology/anatomical area, type of data, dataset, AI method, benchmark measure, and results.

First author (year of publication)	Clinical aim	Pathology/anatomical area	Type of data	Dataset	AI method(s)	Benchmark measure	Results
Trajectory definition							
Villanueva (2018)^174^	Risk assessment for trajectory planning	Neurosurgery	CT MRI	1	GA	Risk Time	2347 –98%
Liu (2017)^175^	Planning stereotactic radiotherapy	Brain tumor	CT MRI	46	SVM	pFDR Time	<0.05% –98%
Valisky (2017)^165^	Discrimination between the STN and SN	Parkinson's disease	CT MRI	46	SVM	pFDR Time	<0.05% –98%
Segato (2019)^176^	Curvilinear DBS	Parkinson's disease	MRI-T1WI DTI CT	10	GA	MSD mSD	+145% +25%
Li (2019)^177^	Computer-assisted planning	Epilepsy	MRI-T1WI CT	10	RF Linear regression	PCC	70%
Vakharia (2019)^178^	Trajectory planning for lasers	Epilepsy	MRI-T1WI	95	RF Linear regression	Risk Length Angle MSD	1.1% 93.5 mm 28.8° 6.7 mm

### C. Intra-operative assistance

**FIG. 7.**
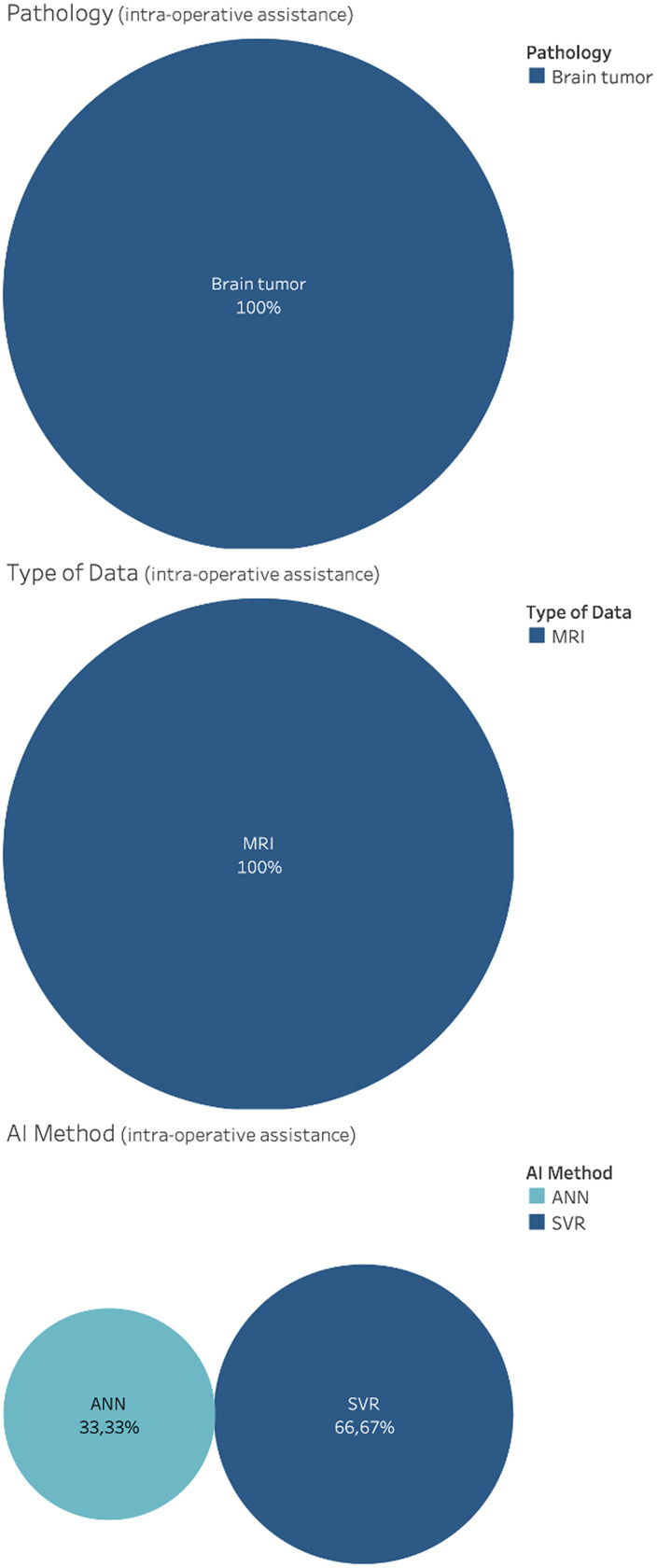
Distribution of general applications for intra-operative assistance, in the brain care literature, related to intra-operative modelling of tissue deformation. From top: pathology, AI method and type of data.

As reported in Table XI, two studies considered AI in intra-operative assistance. This includes modeling of tissue deformation for brain tumor. MRI data ware used as input features for the development of the prediction algorithm. Results of the distributions for pathology, AI methods, and type of data for diagnosis are reported in Fig. 7.

**TABLE XI.** Intra-operative assistance: details on the clinical aim, pathology/anatomical area, type of data, dataset, AI method, benchmark measure, and results.

First author (year of publication)	Clinical aim	Pathology/anatomical area	Type of data	Dataset	AI method(s)	Benchmark measure	Results
Modeling of tissue deformation							
Sharma (2019)^179^	Modeling of tissue deformation	Brain tumor	MRI	4	SVR	PSNR MSE FRDD Acc	17.31% 1240 mm 54% 80%
Tonutti (2019)^180^	Modeling of tissue deformation	Brain tumor	MRI	1	ANN SVR	MSE Error Time	0.11 mm^2^ 0.3 mm 3.1 s

In modeling the tissue deformation for brain tumor surgery, Sharma *et al.*^93,179^ developed a ML approach to detect and model tissue deformation with classification of soft and hard tissues so that the tissues having the risk of future problems can also be recognized. Tonutti *et al.*^180^ presented a real-time soft tissue deformation computation method. A brain tumor was used as the subject of the deformation model. Once trained, the models can predict the deformation of the tumor in real-time with relative positional errors below 0.3 mm. The SVR models perform better than the ANNs, with positional errors for SVR models reaching under 0.2 mm. Thus, given the last results, SVR represents the state-of-the-art approach for modeling the tissue deformation. SVR is a nonparametric technique to perform regression using support vectors through supervised learning. It is based on the kernel machine class of algorithms that uses a similarity function between pairs of data. The goal of the training is to find a function that is as flat as possible, yet deviating from the target data by not more than a chosen value for each point in the training set. Nevertheless, although this method is able to model the tissue deformation using data based on pre-operative anatomical geometry, this means that the deformation model cannot be updated once topological changes occur during the intervention. This is an issue shared by the majority of computational methods in surgery: high accuracy can only be reached if the exact conformation of the anatomy is known at any given time. The proposed method, however, can be expanded to simulate a wide range of surgical scenarios and actions also using intra-operative imaging and knowledge of the preoperative plan.

### D. Postoperative assessment

**FIG. 8.**
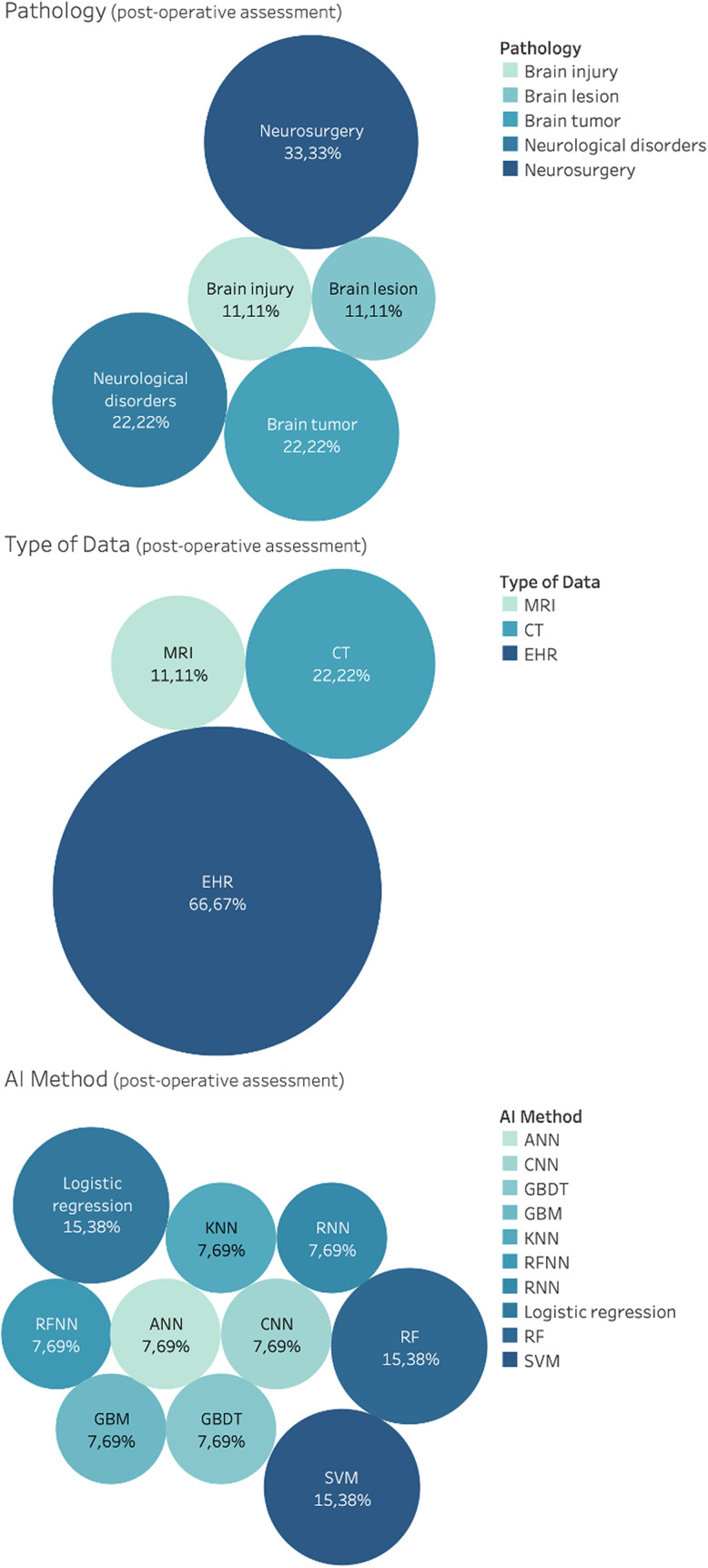
Distribution of general applications for postoperative assessment, in the brain care literature, related to prediction of postoperative patient outcome. From top: pathology, AI method and type of data.

As reported in Table XII, nine studies considered AI in postoperative assessment. This includes prediction of postoperative patient outcomes for brain lesion, brain injury, brain tumor, neurological disorder, and general neurosurgery. CT, MRI, and EHR data were used as input features for the development of the prediction algorithm. Results of the distributions for pathology, AI methods, and type of data for surgical treatment are reported in Fig. 8. DL techniques can be used for testing the informativeness of neurosurgical operative reports for predicting the duration of the postoperative stay in a hospital.^29^ Shabo *et al.*^181^ applied a RNN to the word-embedded texts in EHR. Results prove the potential utility of narrative medical texts as a substrate for decision support technologies in neurosurgery. In two works,^182,183^ six and seven ML algorithms, respectively, were applied to construct Transsphenoidal Surgery (TSS) response prediction models. The ML models showed good discrimination ability and calibration, with the highest levels of accuracy and specificity. The presented models were significantly better than some conventional models.

**TABLE XII.** Prediction assessment: details on the clinical aim, pathology/anatomical area, type of data, dataset, AI method, benchmark measure, and results.

First author (year of publication)	Clinical aim	Pathology/anatomical area	Type of data	Dataset	AI method(s)	Benchmark measure	Results
Prediction of postoperative patient outcomes							
Shabo (2019)^181^	Postoperative hospitalization prediction	Neurosurgery	EHR	…	RNN	MAE	2.8 days
Fan (2019)^182^	TSS response prediction	Neurosurgery	EHR	…	GBDT	AUC	81%
Liu (2019)^183^	CD recurrency prediction	Neurosurgery	EHR	354	RF	AUC	0.78%
Farrokhi (2019)^184^	DBS outcome prediction	Neurological disorders	EHR	501	GBM	AUC Se Sp Acc	…
Merali (2019)^185^	Postoperative outcome prediction	Neurological disorders	EHR	757	KNN RF SVM Logistic regression ANN	AUC Acc Se	70% 77% 78%
Muhlestein (2019)^186^	Postoperative hospitalization prediction	Brain tumor	EHR	41222	29 ML methods	RMSLE	55%
Nie (2019)^187^	Survival prediction	Brain tumor	MRI-T1WI MRI-DTI	68	CNN SVM	Acc	90.66%
Hilbert (2019)^188^	reperfusion prediction	Brain lesion	CT	1301	RFNN	AUC^1^ AUC^2^	71% 65%
Raj (2019)^189^	Mortality prediction	Brain injury	CT	472	Logistic regression	AUC^1^ AUC^2^ Acc	67%–81% 72%–84% 81%–84%

AI can be applied to investigate risk factors and predicting complications in treatments used for the treatment of neurological disorders such as DBS and Dilated Cardiomyopathy (DCM). A work of Farrokhi *et al.*^184^ reports results obtained via supervised learning algorithms achieving high discrimination performance when predicting any complication. Merali *et al.*^185^ applied a supervised ML approach to develop a classification model to predict the individual patient outcome after surgery for DCM. The best performing predictive model used a RF structure and had an average AUC of 0.70, a classification accuracy of 77%, and a sensitivity of 78% when evaluated on a testing cohort that was not used for model training.

AI methods can be used for predicting the inpatient Length of Stay (LOS) after the brain tumor surgery overall survival time. Muhlestein *et al.*^186^ implemented an ML ensemble model to predict LOS with good performance on internal and external validation, which yields clinical insights that may potentially improve patient outcomes. Nie *et al.*^187^ presented a multi-channel architecture of a 3D CNN for DL and a SVM to generate the prediction of the overall survival time. The experimental results demonstrate that this multi-model, multi-channel deep survival prediction framework achieves an accuracy of 90.66%, outperforming all the competing methods.

AI can be applied to investigate risk factors and predicting complications in treatments used for the treatment of brain lesion such as ischemic stroke. Hilbert *et al.*^188^ proposed a DL approach for predicting outcomes of acute ischemic stroke patients using CT angiography images. The model outperformed the models using traditional radiological image biomarkers in three out of four cross-validation folds for functional outcomes (average AUC of 0.71).

AI can also be applied to investigate mortality prediction after traumatic brain injury. Raj *et al.*^189^ used ML-based logistic regression modeling to create two algorithms able to discriminate between survivors and nonsurvivors with accuracies up to 81% and 84%.

Thus, the CNN represents the state-of-the-art approach for prediction of the postoperative patient outcome with an accuracy of 90.66%, outperforming all the competing methods. With a CNN, a hierarchy of appearance features can be synthesized from the low level to the high level in a layer-by-layer manner. The mapping yields a highly sophisticated feature representation for the neuroimages, which is the key advantage of the CNN compared to other machine learning methods. The CNN has shown superior performance in numerous visual object recognition and image classification studies. However, although this method may help to predict postoperative assessment, limited clinical information can bring to a weak clinical model in some cases. To improve the results of this method, it is highly suggested to include in future works other features such as some from pre-surgical imaging, treatment ways, patient statuses before and after surgery, genetic information, and molecular indicators.

## VIII. DISCUSSION

AI algorithms have increasingly caught, in recent years, the attention of many researchers in the neuroscience field. ML, in particular, has been used for finding ways to increase quality and precision of diagnosis and peri-operational decision-making, in order to improve neurosurgical treatments. In this work, a systematic review of recent applications of AI in brain care was presented. Four main categories have been found and analyzed both quantitatively and qualitatively, namely, diagnosis, surgical treatment, intra-operative assistance, and postoperative assessment.

Concerning diagnosis, CNN models are widely adopted. However, despite DL architectures having been demonstrated to be able to achieve excellent results, they present several drawbacks that need to be taken into account. One of the most difficult issues to address is the large amount of data to minimize overfitting and improve performances. However, obtaining them might not be trivial. Several works face this issue by designing proper frameworks that are able to achieve excellent results even using relatively limited amounts of data,^78,191^ training from incomplete data,^192^ and by the adoption of semi-supervised and unsupervised techniques.^190^ These algorithms, indeed, remain a black box in terms of the bases on top of which the predictions are generated from the input data. For this reason, “explainability” will be a crucial part of the development of new algorithms and many research studies follow this direction. To this aim, an interesting alternative is represented by brain connectivity representation of the human brain. Such a kind of data allows us to represent the brain using mathematical models, opening remarkable opportunities to study hidden pathological alterations outside visible objects in conventional images. High performance has been achieved using classical ML and DL models for the diagnosis and interclass classification of several neurodegenerative diseases. An interesting perspective in this sense can be opened by the use of novel graph-based DL approaches, including graph neural networks.^193^ Notwithstanding, as pointed out in related studies,^8^ a major limitation is still the limited sample size, which, however, has started to be overcame by the availability of public datasets; it is worth to mention here The Alzheimer's Disease Neuroimaging Initiative (ADNI)^194^ and The Human Connectome Project.^195^

Concerning surgical treatment, EHR data can be used to select candidates potentially eligible for surgery. As previously mentioned, by Wissel *et al.*,^162^ an electronic health record-integrated NLP application can accurately assign surgical candidacy scores to patients in a clinical setting. In surgical planning, brain structure demarcation may be inaccurate; consequently, the exact detection of the target is difficult, leading to a sub-optimal planning strategy and inadequate clinical outcomes. Interesting applications in surgical treatment concerning the target identification and involving AI approach are obtaining accurate and automatic real-time target detection with intra-operative segmentation and localization of epileptic zones. This work provides neurosurgeons and neurologists with accurate means for automatic patient-specific targeting of the STN and its sub-regions, potentially reducing the need for other approaches that may lengthen the procedure and/or be associated with a higher risk of side effects.^53^ To assist surgeons for a complete planning procedure, AI techniques are exploring the definition of an optimal trajectory giving an alternative to the standard approach such as the graph-based or sampling-based method. In brief, usually, the algorithm aims are to minimize the intra-cerebral trajectory length and drilling angle from orthogonal to skull, while maximizing the distance from critical structures. The use of ML in this context has allowed quantification of hitherto unidentified trajectory parameter combinations to be determined^177^ and the decrease in the time complexity.^174^

Concerning intra-operative assistance and, in particular, intra-operative modeling of tissue deformation, accurate reconstruction and visualization of soft tissue deformation in real time is crucial in image-guided surgery, particularly in augmented reality applications.^180^ The AI approach is able to address the needs of image-guided surgical systems.

In addition, we found that there is emerging interest in the application of AI for postoperative assessment. The accurate prediction of an individual patient's tumor response to treatment is a sort of Holy Grail of oncology.^196^ Indeed, recent discoveries in molecular medicine and improvements in clinical treatments have made it now more important than ever to predict tumor behavior. They have shown that AI methods can predict tumor behavior with greater accuracy than traditional statistical methods.^175^ Mining and advanced analysis of “big data” in brain care provide the potential not only to perform “*in silico*” research but also to provide the predictive model for mortality prediction, postoperative outcome, postoperative hospitalization, and DBS outcomes. “On-demand” access to high-performance computing and large health care databases will support and sustain our ability to achieve personalized medicine. Unfortunately, these increased demands of health care providers create greater risks for diagnostic and therapeutic errors.^197^ Developing a large database of practice guidelines requires knowledge-based technologies to create and maintain them. Ultimately, what is required is also a way for practicing clinicians to access such guidelines quickly, incorporate them into their clinical practices, and then submit their own experiences back to the knowledge base to help improve it.^198^

Although the potential of AI in brain care is promising, in order to observe practical benefits in real-world systems, it is critical to delineate some challenges. Data quality, data inconsistency and instability, and limitations of large size and diversity in support of new studies are some of the major concerns. To this aim, the research community created and populated public repositories and leaderboards to make resources publicly available and submit new results, implicitly dealing with medical-related problems such as validation and legal issues. Kaggle^199^ and Grand Challenge^200^ are concrete examples in this direction. Furthermore, effort is spent to encourage synergy between AI researchers and nontech users (as clinicians and medical experts). In this context, a crucial role is played by web platforms aimed at collaborative learning paradigm that enables research hospitals and institutions to collaborate and develop more robust AI algorithms and collect annotated data. The NVIDIA Clara medical imaging platform,^201^ the Structured Planning and Implementation of New Explorations (SPINE)^202^ project, and the Artificial Intelligence On-Demand Platform and Ecosystem^203^ are some examples.

Although impressive results have been reached, some major hurdles still remain on the road to creation, validation, and deployment of AI in clinical treatment. While AI may produce powerful predictions, this abstraction can lead to hesitation in deploying them. Moreover, the problem of liability emerges about entrusting AI with medical activities. As clinicians make the final decision or interpretation, it can be argued that they have the entire responsibility. To close the gap between clinical practice and AI, we would suggest that future research will concentrate not only on the technological aspects of the design of ML for clinical applications but also on the development of ethical and legal systems for the implementation, validation, and control of AI in clinical care. AI methods should operate in parallel to and applied by clinicians until their accuracy and margin of error are considered appropriate and reasonable, respectively. The rationale such that an error could be considered “appropriate and reasonable” should also be carefully considered as a future challenge. In addition, we believe that, along with many scientists operating in the field, it is strongly recommended that every predictive ML model features the code available to everyone in order to reduce the black-box nature of ML models, and the statistical impediments could be identified and solved, resulting in both a safer and more efficient implementation of AI in brain care. As a final remark, we suggest here that, as DL techniques become more and more effective in solving brain related tasks, a considerable amount of effort should be spent into developing new ways of interpreting such algorithms. Indeed, this study suggests that the primary role of AI in the brain will be to assist experts and clinicians in their duties. For this reason, it is important that researchers do not focus only on algorithm performance but rather also on increasing their trustworthiness. In fact, if there are no consistent and reliable neurobiological variations between two groups of people (e.g., patients vs controls) and even the most advanced machine-learning algorithms would not be able to differentiate on an individual basis between such classes, nevertheless, they would still provide valuable insights.

## IX. CONCLUSION

In this study, a general overview of the current literature on AI methods directly assisting brain care was presented. The use of artificial intelligence techniques is gradually bringing efficient theoretical solutions to a large number of real-world clinical problems related to the brain. Specifically, in recent years, thanks to the accumulation of relevant data and the development of increasingly effective algorithms, it has been possible to significantly increase the understanding of complex brain mechanisms. The researchers' efforts are leading to the creation of increasingly sophisticated and interpretable algorithms, which could favor a more intensive use of “intelligent” technologies in practical clinical contexts.

## AUTHORS' CONTRIBUTIONS

A.S. and A.M. contributed equally to this work.

## Data Availability

Data sharing is not applicable to this article as no new data were created or analyzed in this study.

## NOMENCLATURE

AD: Alzheimer's Disease^4,6–9^
AI: Artificial Intelligence^1–5,7,9–11,23–25^
ANN: Artificial Neural Networks^3,5,9,10^
ASD: Autistic Spectrum Disorder^9^
AUC: Area Under the Curve^5,9,11^
AVM: Arteriovenous Malformation^8^
CAD: Computer Assisted Diagnostic^5^
CLNet: Corrective Learning Network^8^
CMD: Center of Mass Distance^5^
CNN: Convolutional Neural Network^3,6,7,9,11,23^
CT: Computed Tomography^1,2,5,8–11^
DBS: Deep Brain Stimulation^3,4,8–11,15,19,24^
DCM: Dilated Cardiomyopathy^11^
DL: Deep Learning^2,3,5–9,11,23–25^
DNN: Deep Neural Network^3^
DSI: Dice Similarity Index^5,7,8^
DT: Decision Tree^3,5^
DTI: Diffusion Tensor Imaging^2,5^
EEG: Electroencephalography^3,5,10^
EHR: Electronic Health Record^3–5,9–11,24^
FC: Functional Connectivity^2,5^
FCNN: Fully Convolutional Neural Network^8^
FLAIR: Fluid Attenuated Inversion Recovery^2^
fMRI: Functional Magnetic Resonance Imaging^2^
fNIRS: Functional Near-Infrared Spectroscopy^5,10^
FRDD: Fault Rate Dust Detection^7^
GA: Genetic Algorithm^5^
GAN: Generative Adversarial Network^6^
GBM: Gradient Boosting Machine^5^
GNN: Graph Neural Network^3^
GP: Globus Pallidus^9^
GPU: Graphic Processing Unit^2^
HC: Healthy Control^8,9^
HDD: Housdorff Distance^5^
HMM: Hidden Markov Model^7^
HSI: Hyperspectral Imaging^2,5,9^
ICH: Intra-cerebral Hemorrhage^6^
IUS: Intra-operative Ultrasound^2,5,9^
JC: Jaccard Coefficient^5^
KNN: K-nearest Neighbors^5,10^
LITT: Laser Interstitial Thermal Therapy^10^
LOCATE: LOCally Adaptive Threshold Estimation^8^
LOS: Length of Stay^11^
LRP: Layerwise Relevance Propagation^6^
MAD: Mean Absolute Distance^5^
MAE: Mean Absolute Error^5^
MCI: Mild Cognitive Impairment^6,8,9^
MER: Microelectrode Recording^3,5^
MI: Medical Imaging^1^
ML: Machine Learning^1–11,23,24^
MRI: Magnetic Resonance Imaging^1–3,5,7–10^
mSD: min Square Distance^5^
MSD: Mean Square Distance^5^
MSE: Mean Square Error^5,7^
NLP: Natural Language Processing^5,9,24^
NPV: Negative Predictive Value^5^
PCC: Pearson Correlated Coefficient^8^
PET: Positron Emission Tomography^1,2,5^
PPV: Positive Predictive Value^5^
PSNR: Peak Signal-to-Noise Ratio^5,7^
RF: Random Forest^3,5,6,8,10,11^
RMSE: Root Mean Square Error^5^
RNN: Recurrent Neural Network^3,11^
ROC: Receiver Operating Characteristic^5,10^
rs-fMRI: Resting State Functional Magnetic Resonance Imaging^2^
SAE: Sparse Autoencoder^5,8^
SC: Structural Connectivity^2,5^
STN: Subthalamic Nucleus^9,10^
SVM: Support Vector Machines^3,5–11^
SVR: Support Vector Regression^7,10^
TLE: Temporal Lobe Epilepsy^8^
TSS: Transsphenoidal Surgery^11^

## APPENDIX: SYSTEMATIC REVIEW KEYWORDS

Keywords for the systematic review are provided in Table II.
